# Key roles of ubiquitination in regulating critical regulators of cancer stem cell functionality

**DOI:** 10.1016/j.gendis.2024.101311

**Published:** 2024-04-17

**Authors:** Qianqian Guo, Hai Qin, Zelong Chen, Wenzhou Zhang, Lufeng Zheng, Tingting Qin

**Affiliations:** a Department of Pharmacy, The Affiliated Cancer Hospital of Zhengzhou University & Henan Cancer Hospital, Zhengzhou, Henan 450008, China; b Department of Clinical Laboratory, Beijing Jishuitan Hospital Guizhou Hospital, Guiyang, Guizhou 550014, China; c The Affiliated Cancer Hospital of Zhengzhou University & Henan Cancer Hospital, Artificial Intelligence and IoT Smart Medical Engineering Research Center of Henan Province, Zhengzhou, Henan 450008, China; d School of Life Science and Technology, Jiangsu Key Laboratory of Carcinogenesis and Intervention, China Pharmaceutical University, Nanjing, Jiangsu 211198, China

**Keywords:** Cancer progression, Cancer stem cell, E3 ubiquitin ligase, Signalingtransduction, Ubiquitin

## Abstract

The ubiquitin (Ub) system, a ubiquitous presence across eukaryotes, plays a crucial role in the precise orchestration of diverse cellular protein processes. From steering cellular signaling pathways and orchestrating cell cycle progression to guiding receptor trafficking and modulating immune responses, this process plays a crucial role in regulating various biological functions. The dysregulation of Ub-mediated signaling pathways in prevalent cancers ushers in a spectrum of clinical outcomes ranging from tumorigenesis and metastasis to recurrence and drug resistance. Ubiquitination, a linchpin process mediated by Ub, assumes a central mantle in molding cellular signaling dynamics. It navigates transitions in biological cues and ultimately shapes the destiny of proteins. Recent years have witnessed an upsurge in the momentum surrounding the development of protein-based therapeutics aimed at targeting the Ub system under the sway of cancer stem cells. The article provides a comprehensive overview of the ongoing in-depth discussions regarding the regulation of the Ub system and its impact on the development of cancer stem cells. Amidst the tapestry of insights, the article delves into the expansive roles of E3 Ub ligases, deubiquitinases, and transcription factors entwined with cancer stem cells. Furthermore, the spotlight turns to the interplay with pivotal signaling pathways the Notch, Hedgehog, Wnt/β-catenin, and Hippo-YAP signaling pathways all play crucial roles in the regulation of cancer stem cells followed by the specific modulation of Ub-proteasome.

## Introduction

According to the World Health Organization, cancer is the leading cause of premature death, accounting for approximately 30% of noncommunicable disease deaths.^1^ According to the latest statistics from the American Cancer Society and the National Center for Health Statistics, 1,918,030 new cancer cases and 609,360 cancer deaths are expected to occur in the United States by 2022, with approximately 350 deaths per day from lung cancer, the leading cause of cancer death. From 2014 to 2018, the incidence of female breast cancer continued to increase slowly (by 0.5% per year), and the incidence of prostate cancer remained stable, although the incidence of advanced disease has increased by 4%–6% per year since 2011. Moreover, the proportion of diagnoses of advanced prostate cancer has risen from 3.9 percent to 8.2 percent over the past decade.^2^ Currently, clinical cancer treatments mainly include surgery, radiotherapy, chemotherapy, immunotherapy, targeted therapy, and combination therapy. Targeted therapy and immunotherapy are two advanced cancer treatment strategies in the world. Targeted therapy strategies are based on cell-autonomous mechanisms of tumor growth and survival, targeting tumor cells directly. Although these approaches produce clinical responses in most cancer patients with specific gene mutations, due to the high degree of heterogeneity of cancer cells, the lack of persistence in the treatment process leads to tumor metastasis and recurrence.^3^ The heterogeneity of tumor cells makes human cancers all have a subpopulation of cancer stem cells (CSCs) with tumor initiation capabilities, compared with most tumor cell populations.^4^ CSCs are characterized by their ability to self-renew and their potential to differentiate into constitutive tumor cells.^5^ Many articles have reported the discovery of CSCs in various types of tumors, including lung cancer, liver cancer, breast cancer, leukemia, brain cancer, and colorectal cancer.^6^, ^7^, ^8^, ^9^, ^10^ Although genomic instability and epigenetics play a crucial role in CSC function, recent studies have revealed that CSC involvement in translational and post-translational modifications has become a fundamental regulator.^11^^,^^12^ Ubiquitination is a post-translational modification in which the main mechanism is the tagging of substrate proteins by ubiquitin (Ub) molecules to induce their degradation. Thus, the entire Ub proteasome system precisely controls the “quantity” and “quality” of specific proteins to ensure cellular homeostasis. This makes ubiquitination play an important role in the maintenance of CSCs.^13^ However, due to the diversity and complex heterogeneity of CSCs, the current clinical response rate for therapies targeting CSCs remains low, and many scientists are working to develop combinatorial strategies and identify new therapeutic targets, such as searching for novel signaling pathways regulated by CSCs and associated mechanisms of post-translational modification starvation, thus targeting intracellular signaling molecules offers additional opportunities for enhancing anti-tumor immunity.

In this comprehensive review, we will delve into the intricate interplay of protein stability, orchestrated by Ub-related molecular receptors, and its profound implications across various critical processes of CSC progression, including self-renewal, maintenance, and differentiation, highlighting its potential as an effective therapeutic target for anti-cancer drug development.

## Cancer stem cells

**Figure 1:**
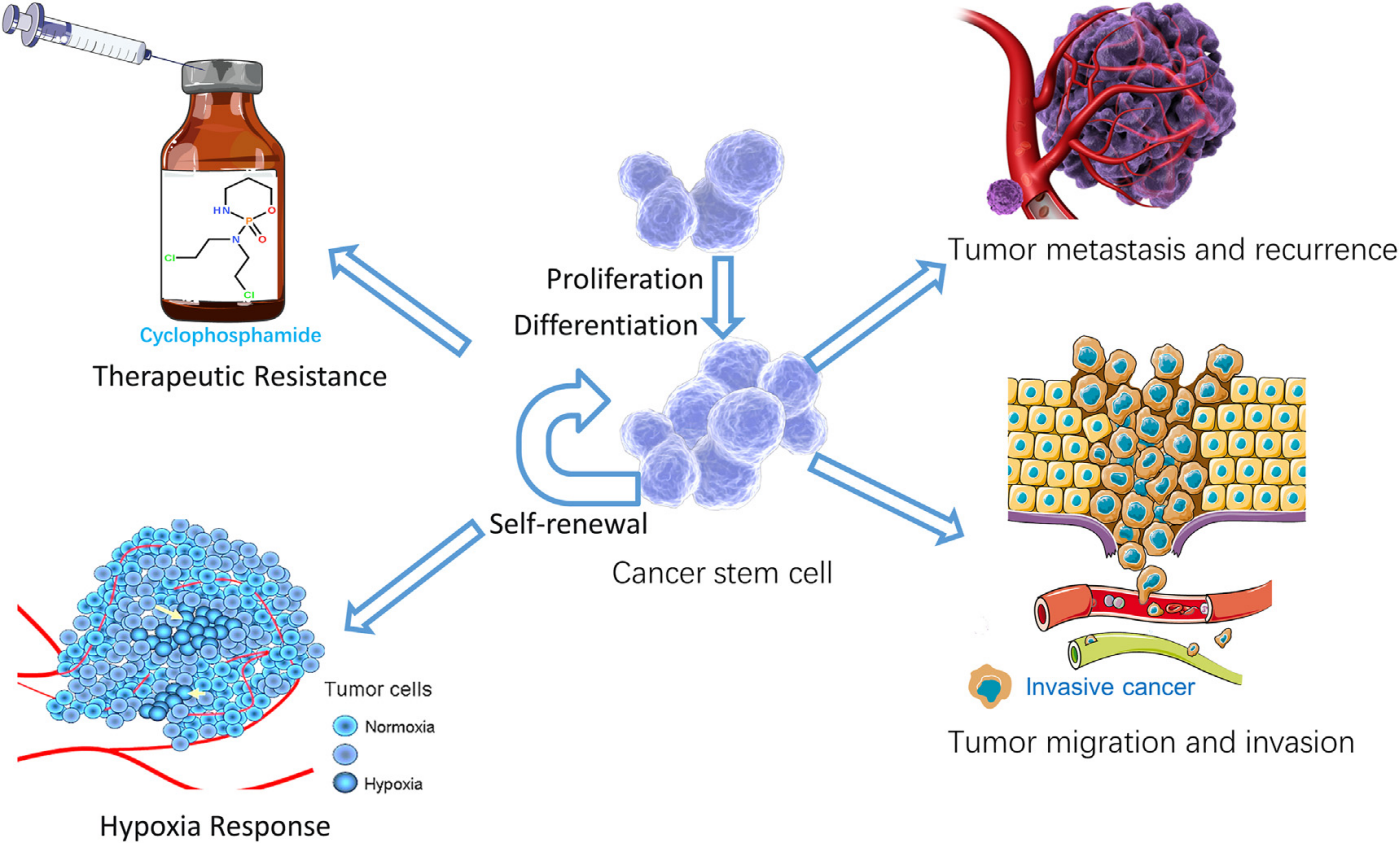
Mechanisms associated with the genesis of cancer stem cell stemness, such as oxidative stress, tumor metastasis and recurrence, hypoxia induction, and tumor invasion.

Reya et al first proposed the theory of CSC in 2001, suggesting that there is a small group of cells with stem cell characteristics in tumor tissue, which is called CSC.^14^ CSCs, like human embryonic stem cells (ESCs), have infinite reproductive capacity, the ability of continuous self-renewal, and multi-directional differentiation potential. The “stem” state of stem cells is the key ability of tumor cells to self-renew, proliferate, and differentiate. Stem cells are found in adult and embryonic tissues and play a vital role in cell regeneration, growth, and embryonic development. Recent studies have identified CSCs as the primary cells for tumorigenesis, recurrence, and metastasis, and many CSCs or CSC-like cells have been identified and isolated in various tumor types.^15^ In recent years, a plethora of research has substantiated that CSCs constitute a distinct subset within the tumor cell population, serving as the fundamental instigators of tumor proliferation, metastasis, recurrence, and the formidable challenge of drug resistance (Fig. 1).^16^ Conventional treatments, while proficient at targeting cells with proliferative potential, regrettably falter in their impact on quiescent CSCs, thus elucidating the perplexing phenomenon of marginal improvements in overall patient survival despite reductions in tumor volume achieved through treatment interventions.^17^ Additionally, owing to the inherent plasticity and pronounced heterogeneity of CSCs, the quiescent CSC population might engender a substantial pool of circulating CSCs, thereby precipitating the failure of clinical interventions.^18^ Notably, CSCs share the same genomic instability and mutation-driven attributes as typical cancer cells, yet they distinctly stand apart from non-stem cells, marked by unique developmental traits spanning epigenetic modifications to variances in gene expression profiles.^19^ After radiotherapy and chemotherapy for any type of tumor, there is a residual body enriched with a small percentage of CSCs.^20^ While CSCs are randomly distributed within tumors, they predominantly present within hypoxic, low pH, and less nutritious environments. This strategic positioning significantly underscores their pivotal roles in instigating tumor initiation, fueling recurrences, and orchestrating the web of metastatic dissemination.^21^ Within the intricate landscape of solid tumors, CSCs are acknowledged as a relatively rare cellular contingent, typically constituting around 1% of total cells in various malignancies such as liver, breast, lung, esophageal, and colorectal cancers.^22^, ^23^, ^24^ In the context of osteosarcoma, their presence is discerned at a range of about 0.1%–1%.^25^ In certain instances, like colon cancer, this proportion escalates to approximately 2%.^26^ Nevertheless, the spotlight intensifies when tumors embark on the treacherous path of metastasis, with CSCs comprising a more substantial 30% fraction in various organs throughout the body. This surge correlates with heightened treatment resistance.^27^ Within the realm of hepatocellular carcinoma, liver cancer stem cells are a small subset of tumor cells with high self-renewal capacity, strong tumor initiation potential, and unlimited differentiation ability. The regulation of cancer stemness in hepatocellular carcinoma involves various mechanisms and factors, such as mitochondrial autophagy, mitochondrial dynamics, epigenetic modifications, tumor microenvironment, and tumor plasticity.^28^ A pertinent example reveals NF-κB, a pivotal inflammatory modulator, intricately involved in reversing the differentiation of tumor-derived hepatocytes to hepatic progenitor cells. Other cytokines, such as TNF-α, IL-6, CCL22, and TGF-β, similarly facilitate this reversal, propelling tumor-derived hepatocytes into a stem/progenitor cell state.^29^ The emergence of breast CSCs, initially isolated from patient-derived xenograft models by Al-Hajj et al in 2003, unveils a unique profile characterized by surface markers CD44^+^CD24^−/low^ and the absence of genealogical markers.^30^ Breast CSCs can lead to breast cancer recurrence and metastasis through different pathways, including heightened expression of adenosine triphosphate-binding transporter proteins, which mitigate drug entry by actively effluxing drugs and bestowing breast CSCs with survival advantages. Modulating ABC transporter expression enhances breast CSC sensitivity to chemotherapeutic agents, as evidenced *in vivo* and *in vitro*.^31^ Furthermore, some classical signaling pathways such as Hippo and Wnt signaling pathways intricately contribute to the stem cell maintenance of CSCs. Ubiquitination emerges as a linchpin process governing the facets of CSC self-renewal, maintenance, differentiation, and tumorigenesis. Notably, studies have unveiled intriguing interactions, for instance, PKMYT1's physical interaction with β-catenin, which stabilizes β-catenin protein and activates Wnt signaling, bolstering non-small cell lung cancer CSC self-renewal.^32^ Additionally, the CSN6-TRIM21 axis has surfaced, spotlighting its role in driving cancer stemness during tumorigenesis. The mechanism involves CSN6 stabilizing OCT1 by down-regulating TRIM21 E3 Ub ligase activity, consequently heightening aldehyde dehydrogenase 1A1 expression and modulating colorectal cancer stemness in an unexpected twist. Quantitative proteomics analyses by Iannis et al revealed discernible differences in protein expression and ubiquitination levels between pluripotent and differentiated stem cells. Intriguingly, core transcription factors including Nanog, Oct4, and Sox2 were identified as ubiquitination targets, hinting at a role for ubiquitination in sustaining stemness and pluripotency within stem cells.^33^^,^^34^ Taken together, these clues collectively underline the intricate web of CSC maintenance, wherein ubiquitination-mediated transcriptional regulatory networks and signaling pathways assume a central role in preserving stemness and pluripotency.

## Ubiquitination and deubiquitination processes

**Figure 2:**
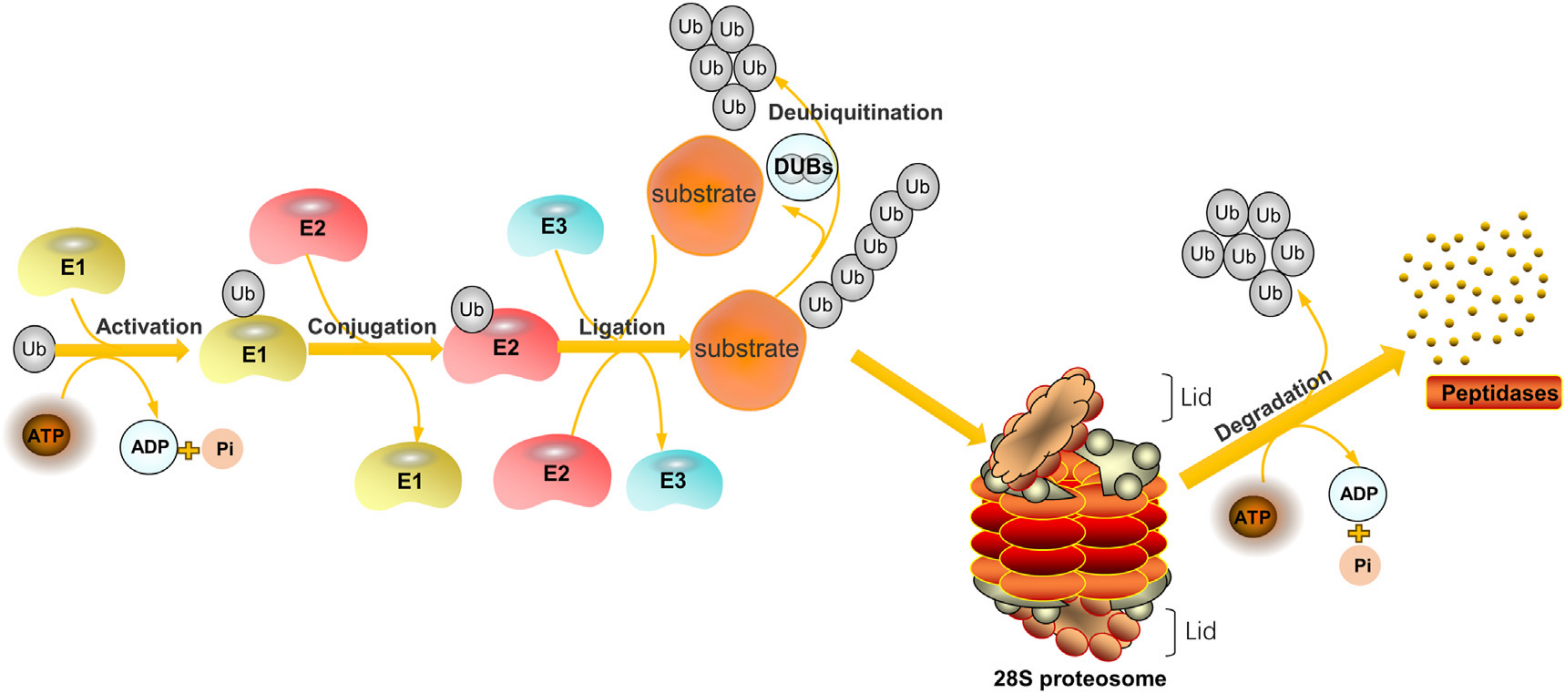
The schematic representation of the Ub-proteasome pathway. Ub, ubiquitin; DUBs, deubiquitinating enzymes.

**Figure 3:**
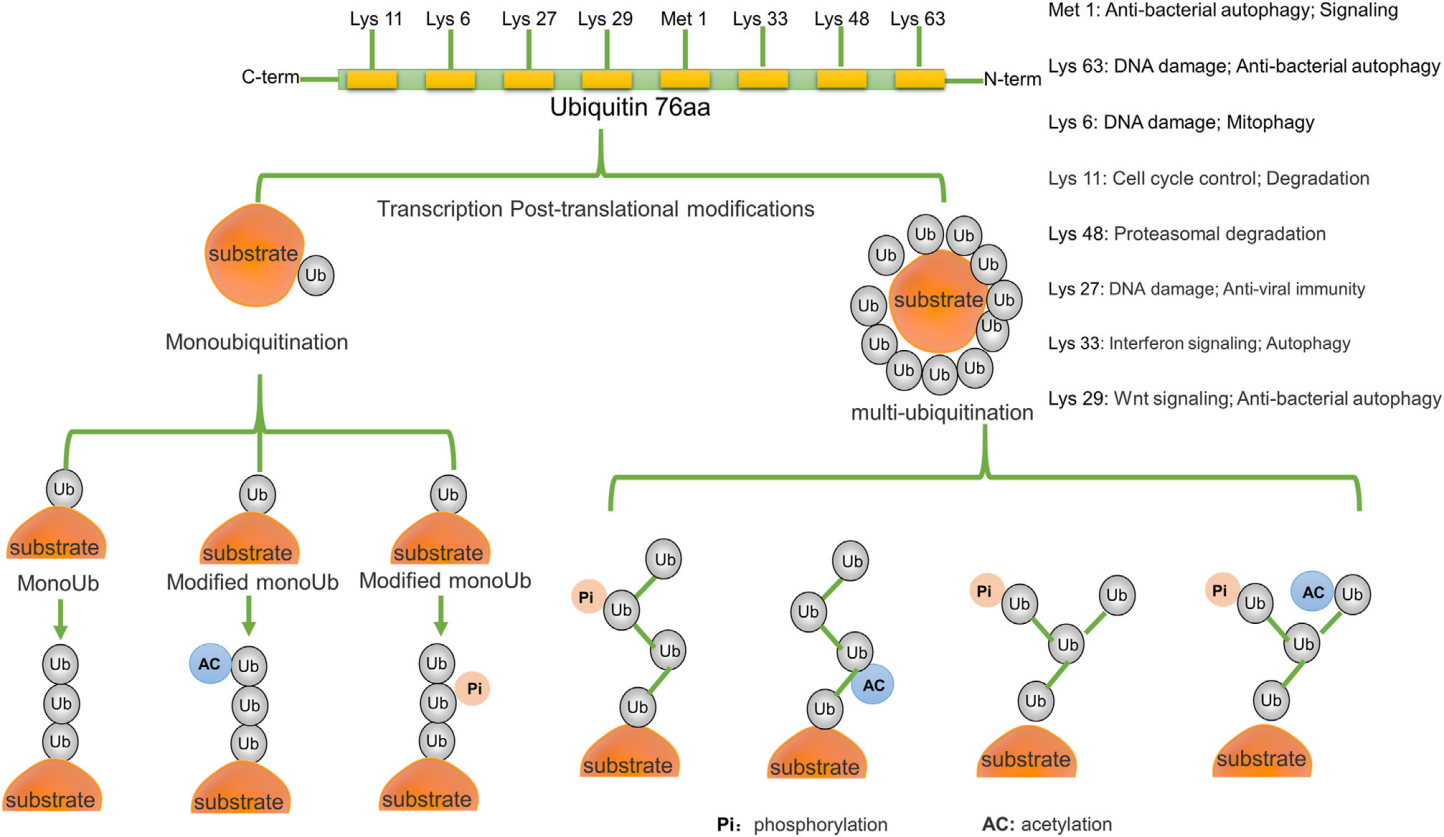
The ubiquitin (Ub) attaches to target proteins in three distinct ways: single attachment, multiple attachment, and chain attachment.

Ubiquitination, a post-translational protein modification, involves a three-enzyme cascade: Ub activating enzyme (E1), Ub conjugating enzyme (E2), and Ub protein ligase (E3). This process connects Ub to substrate proteins through a sequential catalytic pathway. Ub, a compact 76-amino acid protein, forms covalent bonds with other proteins, influencing downstream signal transduction cascades in various biological processes.^35^ Ub modifies proteins through Lys-48 or Lys-11 linked polyUb chains, signaling proteasomal degradation.^36^ The Ub–proteasome system (UPS) regulates diverse physiological functions, including proteasomal protein degradation, protein–protein interactions, DNA repair, gene transcription, kinase activation, and receptor endocytosis.^37^ UPS components include Ub, E1s, E2s, E3s, deubiquitinating enzymes (DUBs), and 26S proteasome.^38^ E1s form Ub adenylate intermediate, providing energy for ubiquitination (Fig. 2). Ub attaches to proteins in single, multiple, or chain forms (*e.g.*, K48, K63), regulating stability, activity, and localization (Fig. 3). Single attachment regulates stability, activity, and localization; multiple attachment occurs when Ub is conjugated to multiple sites, commonly regulating protein function and subcellular localization; chain linkage, including modes like K48, K63, and linear chains, forms a chain-like modification influencing diverse protein functions and destinies.^39^ The recruitment and activation of Ub by E1 is facilitated through the utilization of ATP energy.^40^ E2s are involved in the second step in the ubiquitination modification process, which is the transfer of Ub molecules from Ub-activating enzymes (E1) to substrate proteins.^41^ Moreover, among the three enzymes involved in ubiquitination, there are more than 600 E3 ligases encoded in the human genome which are divided into three families according to different Ub transfer domains,^42^ RING, HECT, and RING-between-RING.^43^^,^^44^ Common E3 Ub ligases include MDM2, SCF complex, Parkin, Cbl, CHIP, RNF20/40, TRIM32, APC/C, NEDD4, and RING1B/RNF2.^45^ Numerous reviews have summarized the key roles played by the TRIM protein family, a subgroup of RNF proteins, in cancer, inflammation, infection, and neuropsychiatric disorders.^46^ The function of E3s in the HECT domain mainly catalyzes the transfer of Ub to the substrate protein through a two-step reaction: the transfer of Ub to the catalytic cysteine on E3, and then the transfer from E3 to the substrate protein. Genetic alterations, abnormal expression, or dysfunction of E3s are associated with the occurrence and development of human cancers.^45^ E6AP, which binds to the oncogenic human papillomavirus-encoded E6 protein, targets p53 for Ub-dependent degradation.^47^ E3 enzymes stand as pivotal constituents within the broader ubiquitination process, exerting precise control over the efficiency and substrate specificity of ubiquitination reactions. To harness the potential of protein ubiquitination, eukaryotic cells have developed an array of Ub ligases, comprising a core set of catalytic units and an assortment of substrate recruitment modules and regulatory elements. These distinctive attributes empower Ub ligases to operate across a spectrum of cellular scenarios, respond to a myriad of intricate cellular cues, and engage in the modulation of a wide range of protein substrates.^48^ Ub ligases play a dual role in the intricate landscape of cancer biology: they can orchestrate the degradation of oncogenes to mitigate their impact or promote the breakdown of tumor suppressor genes, thereby establishing E3s as prospective targets for cancer therapeutics. Notably, discrete domains within E3 Ub ligases assume pivotal roles in facilitating the precise interaction between Ub and its designated target proteins. The E3 Ub ligase collaborates harmoniously with the Ub-activating enzyme E1 and the Ub-conjugating enzyme E2, collectively catalyzing the ubiquitination of a myriad of biologically significant protein substrates destined for recognition by the 26S proteasome. Through this intricate process, targeted degradation is achieved, complemented by an equally crucial role in orchestrating non-proteolytic regulatory mechanisms that fine-tune both function and subcellular localization.

In addition, in the realm of maintaining equilibrium, DUBs emerge as crucial players. These specialized proteases exert an opposing influence, unwinding the protein ubiquitination process. DUBs are a large family of proteases that remove Ub tags from target proteins during proteasomal degradation. Within the intricate tapestry of the human genome, approximately 100 DUBs have been elucidated through studies.^49^ DUBs mainly include Ub C-terminal hydrolase, Ub-specific protease (USP), Machado-Joseph disease protease, OTU, and the recently discovered MIU-containing novel DUB family protease.^50^ DUBs exert their biological main functions through four molecular mechanisms: i) processing of Ub precursors, ii) recycling of Ub molecules during ubiquitination, iii) cleavage of polyubiquitin chains, and iv) reversal of Ub coupling.^51^

The UPS plays a central role in regulating the levels and activities of a variety of proteins and regulating the cell cycle, gene expression, oxidative stress response, cell survival, cell proliferation, and apoptosis. Targeting the UPS has become an attractive avenue for anti-cancer drug development, especially after the approval and success of proteasome inhibitor drugs (bortezomib and carfilzomib).^52^ For the treatment of relapsed or refractory multiple myeloma, a fervent pursuit of improved therapeutic approaches has catalyzed a flurry of studies and clinical trials. These endeavors are meticulously directed toward uncovering novel cancer treatments, focusing on specific components nestled within the ubiquitination pathway. The ultimate objective of these initiatives is to pave the way for more effective interventions, characterized by enhanced efficacy and a reduced burden of side effects.

## The ubiquitination mechanism regulating key molecules in CSCs

**Figure 4:**
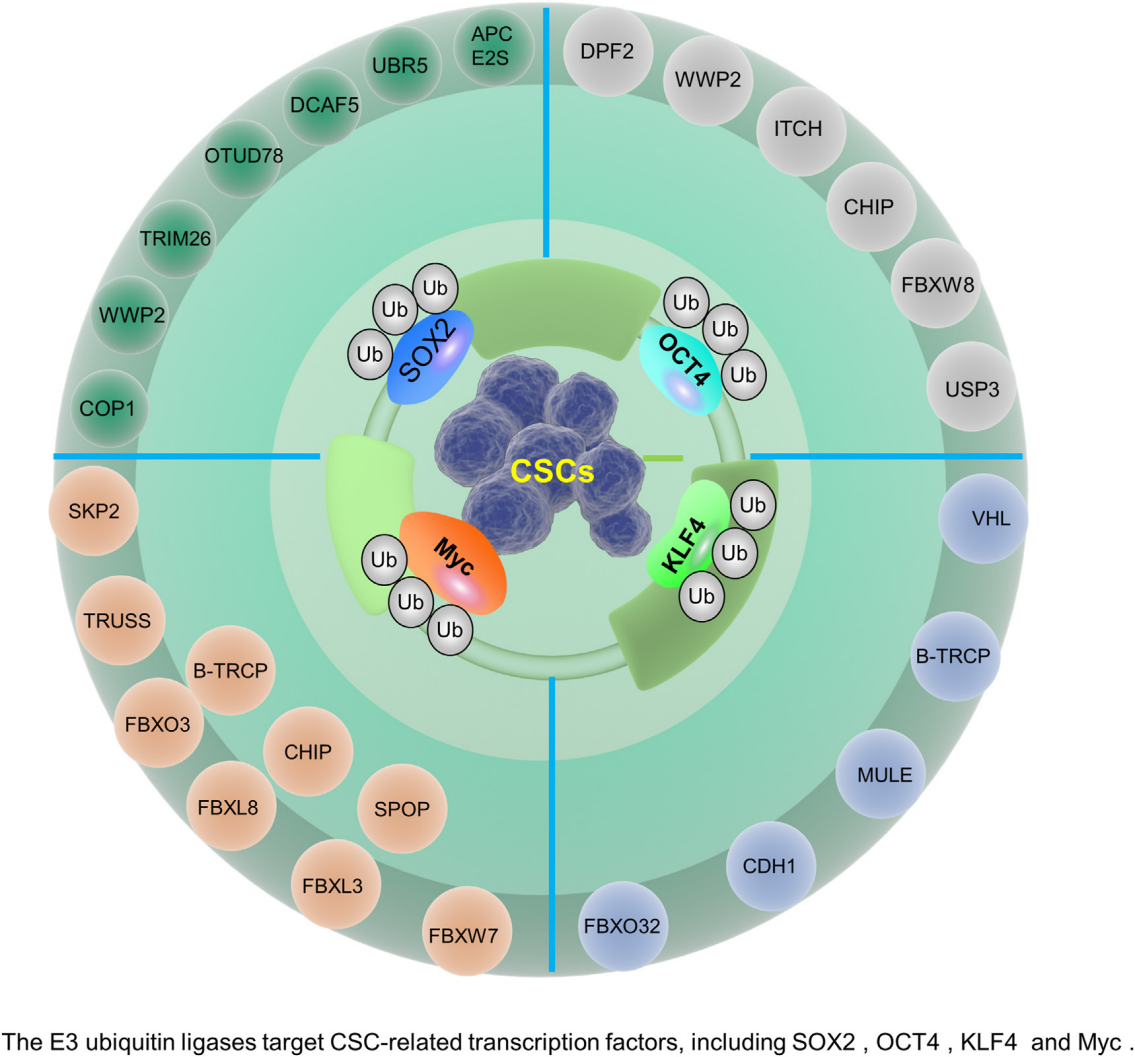
The E3 Ub ligases target CSC-related transcription factors, including SOX2, OCT4, KLF4, and Myc. Ub, ubiquitin; SOX2, SRY-box transcription factor 2; OCT4, octamer -binding transcription factor 4; KLF4, Krüppel-like factor 4.

E3 Ub ligase is a key enzyme in the ubiquitinating enzyme system that links Ub to target proteins, thereby regulating the stability, function, and subcellular localization of the target proteins. Recent studies have shown that E3 Ub ligases play important roles in stem cell maintenance and differentiation, especially in stem cell self-renewal and CSC formation and maintenance. This section will review the molecular mechanisms by which E3 Ub ligases target CSC-associated transcription factors SOX2, OCT4, KLF4, and Myc (Fig. 4).

## Ubiquitination of transcription factor SOX2 in CSCs

Transcription factors constitute a group of proteins with the ability to attach to DNA, holding a pivotal role in governing gene transcription processes. These factors possess the capability to specifically bind to the promoter regions, thereby orchestrating the transcriptional activity of genes through intricate interactions with RNA polymerase and other regulatory protein entities.^53^ Transcription factors are involved in the regulation of gene expression in a spatiotemporal manner by recruiting activators or repressors, interacting with promoters or enhancers, and participating in core transcriptional machinery.^54^ The main biological functions of transcription factors encompass transcriptional activation/repression, histone modifications, nervous system development, and transcription factor network formation.^55^ There is growing evidence that transcription factors regulate multiple cellular processes, including cell proliferation, apoptosis, migration, invasion, metastasis, epithelial–mesenchymal transition (EMT), CSCs, and drug resistance, and play an important role in tumorigenesis and progression.^56^^,^^57^ Dysregulation of transcription factor expression may lead to disruption of the transcription factor network, which in turn affects the expression of multiple genes, thereby promoting tumorigenesis and malignant progression.^58^

In recent years, numerous studies have shown that E3 Ub ligases target many transcription factors for ubiquitination and degradation in human cancers.^59^^,^^60^ These findings underscore the regulatory capacity of transcription factor expression, which in turn governs the onset and intervention of tumorigenesis.^61^ Emerging research has accentuated the paramount role that transcription factors assume in upholding and finely modulating CSCs.^62^ Certain transcription factors like Oct4, Sox2, and Nanog have emerged as definitive CSC signature factors, wielding the capability to sustain CSC self-renewal and versatile differentiation potential.^63^, ^64^, ^65^ Additionally, the regulatory process of CSCs involves certain transcription factors such as c-Myc, KLF4, and STAT3. The dynamic interplay between transcription factors and CSCs is also conspicuous in their sway over proliferation, survival, and metastatic proclivity. Furthermore, distinct transcription factors including Snail, Twist, and Slug have been shown to facilitate the EMT and enhance the metastatic potential of CSCs, thereby promoting tumor invasion and metastasis. Beyond this, other transcription factors like p53, Nrf2, and HIF1 partake in orchestrating the metabolic adaptation and therapeutic resistance of CSCs. This section will delve into the pivotal role of ubiquitination in regulating transcription factors, exploring its implications for tumorigenesis and its potential for intervention in CSCs.

## Mechanism of PI3K-AKT signaling pathway and COP1 E3 ligase governing transcription factors

Transcription factor SOX2 is a highly conserved protein belonging to the SOX family.^66^ Transcription factor SOX2 is indispensable for embryonic development and plays a pivotal role in preserving the pluripotency of embryonic cells as well as various populations of adult stem cells. However, dysregulation of SOX2 expression has been implicated in a spectrum of cancers. Numerous studies have demonstrated that SOX2 exerts a positive influence on cancer cell properties, including proliferation, migration, invasion, and metastasis.^67^ The mounting evidence further suggests that SOX2 operates as a factor bestowing resistance to conventional cancer therapies while maintaining a presence within CSCs.^68^ Several studies have demonstrated that the stability of the SOX2 protein may be regulated by multiple E3 Ub ligases. For example, as an E3 Ub ligase, COP1 plays a crucial role in the regulation of various biological processes by specifically attaching Ub to target proteins and marking them for degradation. These processes include but are not limited to cell cycle control, apoptosis, DNA damage repair, and other physiological functions via targeting p53, c-Jun, p27, and STAT3.^69^ Cui et al reported that the COP1 E3 Ub ligase facilitates the degradation of SOX2 through ubiquitination, thereby inducing neural progenitor cell differentiation. In contrast, OTUD7B champions SOX2's stability by eliminating poly-ubiquitination conjugates (deubiquitination), ultimately propelling neural progenitor cell expression.^70^ Jianfeng Chen et al demonstrated that the PI3K-AKT-SOX2 axis exerts a pivotal role in the development of resistance to R-CHOP (combination of rituximab, cyclophospha-mide, doxorubicin, vincristine, and prednisone) treatment in diffuse large B-cell lymphoma (DLBCL) cells by impeding Ub-mediated SOX2 degradation and enhancing SOX2 stability, thereby preserving the stemness of drug-resistant DLBCL cells. The proposed strategy introduces a pro-differentiation therapy for CSCs. By leveraging the PI3K inhibitor duvelisib (currently undergoing phase III clinical trials), the induction of DLBCL CSC differentiation becomes a feasible goal. This differentiation-promoting therapy, when combined with R-CHOP treatment, emerges as a potent contender against drug-resistant DLBCL cells. The strategy not only reverses drug resistance but also curbs tumor growth, holding promise as a potential cure for DLBCL. Chen and colleagues' work brings forth a pioneering mechanism for spurring CSC differentiation through SOX2 destabilization, thereby complementing the established use of PI3K/AKT inhibitors in cancer therapy.^71^

## The regulatory mechanism of SOX2 by TRIM26/WWP2

TRIM26 and WWP2 are distinct proteins that exhibit both structural and functional resemblances. Both TRIM26 and WWP2 are E3 ligases, possessing E3 ligase activity and regulating protein stability by recognizing substrate proteins and connecting Ub molecules to substrates, thus playing a crucial role in the Ub-protein conjugation system. Studies have demonstrated that SOX2 is highly expressed in glioblastoma and is indispensable for key germinal stem cell (GSC) phenotypes including self-renewal and tumor initiation, demonstrating that proteasome regulation of SOX2 is critical for the maintenance and function of GSCs.^72^ For instance, Diane and colleagues have demonstrated that CDC20-APC may indirectly regulate SOX2 in GSCs through ubiquitination-mediated degradation of the key E3 ligase against SOX2.^73^ Additionally, Tatenda et al employed proteomic techniques to identify TRIM26, an E3 Ub ligase that directly interacts with SOX2 protein. In patient-derived GSCs, the depletion of TRIM26 resulted in a reduction of SOX2 protein levels and an increase in SOX2 polyubiquitination, indicating that TRIM26 plays a role in promoting the stability of SOX2 protein. Therefore, the knockdown of TRIM26 disrupted the gene network associated with SOX2 and resulted in the suppression of self-renewal ability and *in vivo* tumorigenicity across multiple GSC lines. Mechanistically, TRIM26 stabilizes the SOX2 protein by directly inhibiting the interaction between SOX2 and WWP2 through its C-terminal PRYSPRY domain (independently of its RING domain), ultimately confirming WWP2 as a genuine E3 ligase for SOX2 in GSCs.^74^ An additional investigation has unveiled a distinct facet, showcasing the E3 ligase WWP2's targeted interaction with K119-methylated SOX2 via its HECT domain. This interaction effectively ushers in the ubiquitination of SOX2. In the intricate orchestration of its regulatory framework, SOX2 can undergo mutually exclusive modifications, namely, methylation at K119 by Set7 or phosphorylation at T118 by AKT1. These modifications contribute significantly to its regulatory mechanisms. Notably, K119 methylation operates as a “methyl degron”, serving to recruit the E3 ligase WWP2 and, consequentially, instigate SOX2's degradation. Conversely, T118 phosphorylation plays a contrasting role, stabilizing SOX2 through the inhibition of K119 methylation.^75^ These explorations collectively underscore the integral role played by methylation and phosphorylation switches in meticulously modulating SOX2 protein stability. This fine-tuning extends further to shape its pivotal function in both preserving pluripotency and expediting the differentiation of ESCs.

## L3MBTL3 and CRL4^DCAF5^ Ub ligases are involved in the regulation of SOX2 protein degradation

Lethal (3) malignant brain tumor-like protein 3 (L3MBTL3), a member of the L3MBTL family, functions as an E3 Ub ligase and is associated with malignant brain tumors. L3MBTL3 is believed to function as a transcriptional repressor that modulates gene expression by impeding the transcription process of genes. The primary mechanism employed by L3MBTL3 involves obstructing or diminishing the binding of transcription factors through specific chromosomal sites, including methylation and phosphorylation sites within chromatin, thereby resulting in attenuated transcriptional activity of associated genes.^76^ The intricate machinery of the CRL4^DCAF5^ (Cullin-RING E3 ligase 4 with DDB1-CUL4 associated factor 5) complex serves as an E3 Ub ligase, orchestrating the intricacies of ubiquitination and emerging as a central player in the precise orchestration of targeted protein degradation and cellular regulation. This complex engages in strategic interactions with transcriptional complexes, thereby wielding the power to finely modulate gene expression. This modulation is achieved through a spectrum of mechanisms, ranging from the modification of chromatin structure to the alteration of chromatin accessibility, all the way to the intricate task of regulating the stability of pivotal transcription factors.^76^ Zhang et al previously discovered that the SET7 methyltransferase monomethylated lysine residues 42 and 117 of the SOX2 protein, thereby facilitating its hydrolysis. Conversely, LSD1 and PHF20L1 acted on methylated Lys-42 and Lys-117 to inhibit SOX2 protein hydrolysis.^77^ Their recent study has once again demonstrated that the direct binding of L3MBTL3 to methylated SOX2 protein results in the recruitment of CRL4^DCAF5^ Ub E3 ligase, which subsequently targets SOX2 protein for Ub-dependent proteolysis.^78^

## Other E3 Ub ligases participate in the regulation of SOX2 protein degradation

SOX2 gene amplification has emerged as a potent driver in various malignancies, including lung cancer, gastric cancer, colorectal cancer, and esophageal cancer.^79^, ^80^, ^81^, ^82^ In a distinct study, the spotlight turned to the E3 Ub ligase UBR5, which displayed a nuanced interaction with SOX2 in esophageal cancer cells. This interaction initiated the ubiquitination process, ultimately culminating in the degradation of SOX2. Notably, UBR5 was highlighted as a pivotal Ub E3 ligase orchestrating SOX2 degradation through specific ubiquitination at lysine 115. Curiously, the intricate interplay deepened with the revelation that phosphorylation of SOX2 at threonine 116 by AKT played a role in SOX2 stabilization, effectively curtailing its interaction with UBR5.^83^ Underpinning these dynamics, AKT assumed a pivotal role in preserving SOX2 stability across diverse esophageal squamous cell carcinoma cell lines. Consistent with earlier reports, inhibition of AKT signaling manifested in the down-regulation of SOX2 protein expression in non-small lung cancer cells, while conversely, Akt spurred Sox2 expression in hippocampal neural progenitor cells.^84^^,^^85^ Notably, the AKT inhibitor MK2206 exercised a pronounced impact on SOX2 expression within all evaluated SOX2-positive esophageal squamous cell carcinoma cell lines, notably hampering tumor sphere formation.^83^ In a different arena, MSX2, a key regulator in the differentiation of human pluripotent stem cells, plays a crucial role in the development of the cranial vault, hair follicles, teeth, and mammary glands.^86^^,^^87^ Recent findings underscored MSX2's capacity for transcriptional repression over SOX2 expression.^88^ Further intrigue stemmed from the revelation that FBXW2, acting as an E3 ligase, formed a complex interaction with MSX2, thereby inducing the ubiquitination of MSX2, paving the way for its subsequent proteasomal degradation. This intricate process, in turn, led to the accumulation of SOX2. Noteworthy is the link between SOX2 overexpression and tamoxifen resistance in MCF7 breast cancer cells, where MLN4924 treatment was demonstrated to induce MSX2 accumulation through FBXW2 inhibition, ultimately suppressing SOX2 expression.^89^^,^^90^ This mechanism contributes to the attenuation of stem cell properties in breast cancer cells and the inhibition of tamoxifen resistance.

Although SOX2 itself, as a transcription factor, is not an optimal target for small-molecule therapy, this study posits the targeting of the associated E3 ligase as a promising strategy for treating cancers driven by aberrant SOX2 expression. The identification of specific E3 ligases involved in SOX2 regulation opens avenues for targeted therapeutic strategies. Inhibition or modulation of these E3 ligases presents a promising approach to disrupt SOX2-mediated oncogenic pathways. This could potentially overcome drug resistance, inhibit tumor growth, and attenuate stem cell properties in various cancers. These insights lay the groundwork for future studies aiming to unravel the complexities of transcription factor regulation and exploit them for precision cancer therapies. Taken together, these insights propel future analyses into SOX2's transcriptional targets, promising to elucidate the intricate mechanisms underpinning SOX2-mediated invasiveness, particularly within the realm of CSCs.

## Ubiquitination of Oct4 transcription factors in CSCs

OCT4 is a POU5F1 gene, which belongs to the DNA-binding protein POU family and is encoded by the POU structure (Pit1, Oct1/Oct2, and Unc86). It is located on chromosome 6p21 of the human genome.^91^ Bioinformatic analyses have parsed the nucleotide sequence of the POU5F1 genome, approximating its length at 6 kb. This encompassing structure comprises five exons (E1–E5) interwoven with four introns.^92^ In various human cancer types, the transcription of POU5F1P1 and POU5F1P5 RNA may modulate the activity of the POU5F1 gene, thereby potentially contributing to carcinogenesis.^93^ POU5F1 is predominantly expressed in pluripotent cells, such as the inner cell mass of mammalian blastocysts (early embryos), ESCs, embryonal carcinoma cells, embryonic germ cells, and CSCs.^94^^,^^95^ The relevance of Oct4 deepens as it not only serves as a marker but also assumes a significant role in preserving the stemness of cancer cells.^64^ The expression of OCT4 is positively correlated with the pluripotent properties of stem cells and plays a crucial role in regulating the early stages of mammalian embryogenesis. As it pertains to mouse ESCs, the mRNA levels of Oct4 emerge as key architects in maintaining pluripotency while orchestrating the trajectory of differentiation across trophoblast, primitive endoderm, and mesoderm lineages.^96^ Unveiling a new layer, post-translational modifications of OCT4 establish their primacy in governing its functionality, with potential therapeutic implications spanning a spectrum of diseases.

## The E3 Ub ligase CHIP/WWP2 regulates the stabilization of OCT4 protein

As a member of the Ubox family, CHIP functions as an E3 Ub ligase that exerts dual roles in tumorigenesis by either suppressing or promoting tumor growth. It achieves this by selectively targeting specific substrates for lysosomal or proteasomal degradation, thereby regulating cellular homeostasis.^97^ Xu et al demonstrated a positive correlation between low expression of CHIP E3 ligase in thyroid cancer tissues and cells and improved survival in thyroid cancer patients. Additionally, they found that CHIP directly interacts with OCT4 protein, leading to ubiquitination of Oct4 and reduced stability of OCT4 in thyroid cancer cells. Moreover, the overexpression of CHIP resulted in a reduction in thyroid cancer spheroid formation ability, aldehyde dehydrogenase activity, and stemness marker expression levels. Conversely, the knockdown of CHIP had the opposite effect on thyroid cancer cells.

Mechanistic studies have demonstrated that CHIP inhibits the expression of Oct4 by inducing its ubiquitination, thereby attenuating the stemness of thyroid cancer cells.^98^ Similarly, Cho et al discovered a significant down-regulation of the CHIP E3 Ub ligase expression in breast CSCs. They discovered a direct interaction between CHIP and OCT4, resulting in reduced stability of OCT4 and diminished breast CSC properties.^99^ Mechanistically, CHIP ubiquitinates OCT4 at lysine 284 and the overexpression of CHIP does not lead to degradation of mutant OCT4_K284R. The proliferation and side population of breast cancer cells were increased by the overexpression of mutant OCT4_K284R. Excitingly, the overexpression of mutant OCT4_K284R (1 × 10^3^) in breast cancer cells xenografted into mice resulted in a significant increase in tumor burden. These findings strongly suggest that CSCs exhibit reduced CHIP expression and that the amount of OCT4 is critical for maintaining their stemness.^99^ Additionally, human WWP2 is a WW domain-containing E3 Ub-protein ligase 2, which was initially identified by Pirozzi et al during a screening for proteins containing WW domains. WWP2 comprises four consecutive WW domains, a HECT domain at the C-terminus that is associated with Ub protein ligase activity, and an N-terminal C2-like domain which is a characteristic feature of a large class of proteins.^100^ Xu and his team have demonstrated that OCT4 specifically interacts with WWP2 both *in vitro* and *in vivo*. Their findings serve to demonstrate several significant points: (i) endogenous OCT4 in human ESCs can be subjected to modification by Ub; (ii) WWP2 enhances the ubiquitination and degradation of OCT4 protein through the 26S proteasome in a dose-dependent manner; and (iii) down-regulation of WWP2 expression significantly elevates the levels of OCT4 protein in undifferentiated human ESCs and embryonal carcinoma cells.^101^ Mechanistically, WWP2 functions as a catalyst for the polyubiquitination of OCT4. This action specifically takes place through a process called lysine 63 ligation, where the attachment of multiple Ub molecules is orchestrated. It is intriguing to note that WWP2 also exerts control over its own ligase activity in a similar manner. Notably, WWP2 contributes to the controlled degradation of both auto-ubiquitinated molecules as well as OCT4 within ESCs. This self-regulatory process aids in maintaining a balanced cellular environment.^102^ It is expected to demonstrate whether WWP2-mediated modulation on OCT4 protein holds critical effects on CSCs in the future.

### DPF2, ITCH, and FBXW8 E3 Ub ligases regulate OCT4 protein stabilization

DPF2, also known as ubi-d4/requiem (REQU), contains dual plant homeo domain finger (PHD) finger structural domains that are commonly associated with chromatin modification. The PHD finger structural domains can interact with histone-modifying enzymes, including methyltransferases and acetyltransferases, to regulate the structure and function of chromatin 103. Therefore, it is possible that DPF2 plays a role in processes related to chromatin modification and acts as a regulator for gene expression and chromatin status. In recent years, an increasing number of studies have revealed the involvement of PHD finger proteins in the process of ubiquitination and subsequent degradation of target proteins, suggesting their potential E3 activity.^104^^,^^105^ DPF2 functions as an E3 ligase that specifically targets OCT4 proteins, thereby regulating their nuclear distribution. DPF2 directly interacts with OCT4 and depletion of DPF2 leads to increased protein levels and enhanced stability of OCT4 in P19 mouse embryonal carcinoma cells and human ESC H19 cells.^106^ DPF2 overexpression facilitates the polyubiquitination and subsequent degradation of Oct4 protein, thereby promoting its hydrolysis.^107^ Mechanistically, DPF2 catalyzes the assembly of polyubiquitin chains on OCT4 through K6, K48, and K63 linkages, with a higher efficiency observed for K48 linkages. Both anti-His antibody and anti-OCT4 antibody can unequivocally detect the ubiquitination of OCT4 via K6 linkage DPF2.^106^^,^^107^

ITCH, like DPF2, functions as an E3 ligase and plays diverse roles in cellular processes such as protein degradation, immunomodulation, cell cycle regulation, and tumor suppression. ITCH regulates the ubiquitination of various immune-related proteins, including signaling molecules, transcription factors, and receptor proteins, thereby modulating the signaling pathways and immune responses of immune cells.^108^ ITCH can interact with OCT4, leading to an increase in its ubiquitination and regulation of protein stability. This intricate interplay becomes even more significant as ITCH-mediated hydrolysis takes effect on the OCT4 protein, assuming a pivotal role in the governance of self-renewal and the induction of pluripotency in ESCs. Notably, a reduction in the expression of Itch significantly undermines the defining characteristics of ESCs, underscoring its significance in maintaining their distinct properties. Strikingly, ITCH also contributes to the amplification of OCT4's transcriptional activity within ESCs.^109^ In another study, Kap1 was unveiled as an OCT4-binding protein, exerting an inhibitory influence on Itch-induced OCT4 ubiquitination. This dynamic, in turn, bolsters the stability of OCT4. This regulatory mechanism assumes a pivotal role in processes like somatic cell reprogramming and the self-renewal of ESCs.^110^

FBXW8 is also classified as one of the E3 Ub ligases, which functions as a crucial component within the SCF Ub ligase complex. As an F-box protein in the SCF complex, FBXW8 possesses the ability to interact with target proteins and facilitate their ubiquitination through catalyzing Ub ligation. This ultimately contributes to the targeted proteins undergoing ubiquitination. The primary biological functions of FBXW8 include regulation of the cell cycle, degradation of proteins, and modulation of multiple signaling pathways.^111^ Bae et al demonstrated that FBXW8 has been identified as a regulator of OCT4 through its involvement in proteasomal degradation in ESCs. OCT4 is phosphorylated at Ser 347 by c-Jun N-terminal kinase 1/2, leading to negative regulation of OCT4's transcriptional activity. Inhibition of Ser 347 phosphorylation of OCT4 hinders the differentiation process of mouse ESCs induced by withdrawal of leukemia inhibitory factor. Mechanistic studies further indicate that the OCT4 S347A mutation enhances the stability of OCT4 protein by impeding Ub-mediated protein degradation. Importantly, FBXW8 interacts with and regulates the protein accumulation of OCT4, while phosphorylation of OCT4 at Ser 347 is essential for achieving ubiquitination by FBXW8 and somatic reprogramming.^85^ Taken together, FBXW8 plays a pivotal role in ESCs; however, further investigation is warranted to elucidate the regulatory mechanism of FBXW8 in CSCs through targeted modulation of OCT4.

The Ub-mediated regulation of OCT4 by various E3 ligases adds a layer of complexity to our understanding of stem cell dynamics and tumorigenesis. Deciphering the intricacies of these regulatory mechanisms not only advances our knowledge of pluripotency maintenance but also opens avenues for developing targeted therapeutic interventions in cancer, with potential implications for precision medicine and improvement of treatment outcomes. Targeting specific E3 ligases involved in OCT4 degradation could be explored to modulate stemness properties in CSCs, providing a nuanced approach to disrupt cancer progression. However, the intricate relationships and context-dependent roles of E3 ligases in diverse cellular processes necessitate further investigations to unravel their precise contributions in different cancer types and stages.

### Ubiquitination of c-Myc transcription factors in CSCs

C-Myc is a transcription factor that exerts significant influence on biological processes, including but not limited to cell proliferation, cell cycle regulation, cell differentiation, apoptosis, and tumorigenesis.^112^ Mechanistically, c-Myc regulates the activity of tumor-related signaling pathways such as Wnt/β-catenin, Ras/MAPK, and PI3K/Akt to promote tumorigenesis and progression.^113^ Moreover, c-Myc plays a pivotal role in CSCs, exerting its mechanism of action primarily through the following aspects: i) Maintenance of stem cell properties: One core facet of c-Myc's engagement in CSCs involves activating the expression of cardinal stem cell-associated genes, such as Oct4, Sox2, and Nanog. This deliberate orchestration serves to perpetuate the self-renewal and proliferative capabilities inherent to stem cells.^114^ ii) Dampening differentiation: Within CSCs, c-Myc asserts its authority by stifling the process of cellular differentiation. Its overexpression curbs the expression of genes linked to differentiation, such as C/EBPα and GATA1, effectively hampering the maturation journey of CSCs.^115^ iii) Empowering cell invasion and metastasis: The capacity of c-Myc extends to enhancing the invasive and metastatic prowess of CSCs. By activating transcription factors like Slug and Snail, c-Myc propels the EMT, thereby fostering CSCs' ability to invade and disseminate.^116^ iv) Apoptotic regulating: Another role carved out by c-Myc is its governance over the life-or-death decision in CSCs. Through its orchestration, overexpression of c-Myc quells the expression of apoptosis-associated genes, such as Bax and Bad. This strategic modulation bolsters the survival capacity of CSCs.^117^ These multifarious mechanisms collectively vest c-Myc with a pivotal regulatory stature concerning the intrinsic biological attributes of CSCs and the intricate landscape of tumor development. Numerous studies have demonstrated that the expression of c-Myc is subject to regulation by a variety of E3 ligases. Thus, our forthcoming exploration will delve into the realm of Ub E3 ligase-mediated proteasomal degradation, unmasking the intricate dance that marks the fate of c-Myc.

### The FBXL family modulates the stability of c-Myc protein

The FBXL family is a large group of proteins that contain F-box domains and leucine-rich repeat sequences. These proteins are typically components of the SCF Ub ligase complex, which plays a crucial role in regulating substrate protein ubiquitination and degradation.^118^ By regulating the stability and function of substrate proteins, the FBXL family plays a crucial role in maintaining cellular homeostasis, modulating biological processes, and contributing to tumorigenesis.^119^ As an illustration, Cry2 functions as a scaffolding protein to assemble an active SCF-FBXL3 complex that interacts with c-MYC, thereby facilitating the ubiquitination and degradation of c-MYC. Interestingly, deletion of Cry2 stabilizes c-Myc and prevents the interaction between CRY2/FBXL3 and phosphorylated T58 residue on c-Myc. Consequently, the absence of CRY2 triggers a shift in the equilibrium, leading to the development of Myc-driven lymphoma in mice.^120^ Furthermore, Guo et al initially identified a direct regulatory relationship between miR-181d, c-myc, and CRY2/FBXL3 expression, with overexpression of miR-181d resulting in a significant reduction of CRY2 and FBXL3 protein levels. CRY2 and FBXL3 exhibited a negative correlation with the expression levels of miR-181d, which facilitates aerobic glycolysis by safeguarding c-myc from degradation mediated by FBXL3 and CRY2. This mechanism is accountable for the growth and metastasis of colorectal cancer.^121^ Moreover, Skp2 has recently been demonstrated to participate in the degradation of c-Myc through its interaction with Myc box II (and the HLH-Zip region).^122^ However, MB I is also implicated in the degradation of c-Myc, and phosphorylation of Thr-58 and Ser-62 in MB I is believed to primarily regulate the stability of c-Myc. Intriguingly, the majority of cancer-associated mutations within c-MYC predominantly target these residues or their adjacent counterparts.^123^ Meanwhile, it has been evidenced that the c-Myc fragment (LPTPPLSP) present within MB I corresponds to the CPD sequence recognized by Fbw7.^124^ Recent work by Yada et al has illuminated the interaction between F-box protein Fbxw7 and c-Myc in a manner contingent on the phosphorylation state of MB I. This interaction leads to the destabilization of c-Myc. Notably, while wild-type Fbxw7 expedites c-Myc turnover, Fbxw7 mutants devoid of the F-box domain display delayed transformation.^124^ Several studies have reported that Fbxw7 is capable of targeting c-Myc for ubiquitination and subsequent degradation.^125^ Fbxw7 wields a regulatory role within the GSK3 phosphorylation-driven degradation of c-Myc. Specifically, GSK3 phosphorylation at Thr-58 orchestrates a modulation in the interaction between Fbxw7 and c-Myc. This pivotal interaction alteration facilitates the proteolysis of c-Myc.^125^^,^^126^ In the context of ESC differentiation, the autophagy-associated 5 protein interacts with c-Myc, recruiting Fbxw7 to facilitate the ubiquitination-mediated degradation of c-Myc, which is a prerequisite for ESC differentiation.^127^ Within the macrophage and tumor immune microenvironment, Fbxw7 was observed to suppress M2-like polarization of tumor-associated macrophages, thereby limiting tumor progression. Fbxw7 exerts this influence through mediating c-Myc degradation via the UPS. Mechanistically, Fbxw7 intervenes in the polarization of M2-like tumor-associated macrophages by orchestrating c-Myc's degradation.^128^ Notably, c-Myc features as a notable substrate of Fbxw7 in the context of maintaining ESC equilibrium and aiding somatic cell reprogramming. The orchestration of pluripotency hinges on Fbxw7's capability to target c-Myc's stability.^34^ In triple-negative breast cancer, NRG1 is predominantly expressed and promotes the expression of HER2 receptors, thereby facilitating the growth and metastasis of breast cancer.^129^ Fra-1 is a subunit of the AP-1 transcription factor and an overexpressed transcription factor in triple-negative breast cancer that functions as a metastasis coordinator. The transcriptional regulation of Fra-1 by NRG1 is facilitated through ERK1/2-induced recruitment of c-Myc to the promoter region of Fra-1. Moreover, the E3 ligase Fbxw7 targets c-Myc for ubiquitination and degradation, with NRG1 expression and ERK1/2-mediated phosphorylation of Fbxw7 regulating this process by inducing c-Myc dissociation and nuclear import.^130^ Moving forward, FBXL8 functions as an adaptor subunit of the SCF Ub ligase complex, specifically targeting c-Myc for selective degradation through its F-box and leucine-rich repeat domains. SCF-Fbxl8 binds to and ubiquitinates c-Myc independently of phosphorylation, indicating its regulation of a distinct pool of c-Myc from that regulated by SCF-Fbxw7. The deletion of FBXL8 resulted in an increase in c-Myc protein levels, protein stability, and cell division, while the overexpression of FBXL8 led to a decrease in c-Myc protein levels.^131^ Within this intricate context, FBXL16 is a less-studied member of the F-box protein family. FBXL16 enhances the levels of proteins targeted by SCF-E3 ligase, including C-MYC, β-catenin, and SRC-3.^132^ FBXL16 stabilizes C-MYC by counteracting Fbxw7-mediated ubiquitination and degradation of C-MYC. The N-terminal F-box structural domain of FBXL16 interacts with SKP1, while its C-terminal leucine-rich repeat sequence structural domain binds to the substrate C-MYC. This dual domain involvement is pivotal in FBXL16's stabilization of C-MYC. FBXL16's ability to elevate oncoproteins C-MYC and SRC-3 aligns with its role in fueling cancer cell growth, migration, and colony formation in soft agar.^132^

### The stability of the c-Myc protein is regulated by SPOP/HUWE1

SPOP is recognized as a tumor suppressor gene, and its mutation or abnormal expression has been linked to the initiation and progression of a diverse array of malignancies.^133^ The SPOP protein functions as an E3 Ub ligase adaptor, facilitating the ubiquitination and subsequent degradation of substrate proteins by interacting with other Ub ligase complexes.^134^ Mutations in SPOP can lead to the loss or alteration of its function, which impacts substrate protein degradation and cellular biological processes, ultimately promoting tumor cell growth and metastasis.^135^ The involvement of SPOP extends to the regulation of degradation for various substrate proteins, encompassing transcription factors, signaling pathway proteins, and cell cycle proteins. Additionally, SPOP is frequently dysregulated through somatic mutations or mRNA down-regulation, indicating its crucial role as a tumor suppressor in prostate adenocarcinoma. *In vitro* expression of SPOP results in physical interaction with c-MYC protein, leading to the promotion of c-MYC ubiquitination and subsequent degradation.^136^ Genomic enrichment analysis revealed that the genes induced by Myc were significantly enriched in transcriptomic features associated with SPOP. Additionally, it has identified c-MYC as a novel and authentic substrate of SPOP and provided insights into the frequent inactivation of SPOP in human prostate adenocarcinoma.^136^ The results of this current study harmonize with the notion that the LINC01638 lncRNA sets in motion MTDH-Twist1 signaling by curbing SPOP-mediated degradation of c-Myc in the context of triple-negative breast cancer. In response, c-Myc assumes the role of an activator for MTDH expression through transcription, subsequently triggering the activation of Twist1 expression. This orchestrated sequence culminates in the induction of EMT.^137^ Functioning as an E3 ligase, HUWE1 assumes a central role in governing several pivotal regulators of the cell cycle, notably through the intricacies of ubiquitination and subsequent degradation. Its reach notably extends to the degradation of cyclin-dependent kinase regulators, including p53, p21, and p27.^138^ Beyond these, HUWE1 wields sway over an array of other cell cycle-linked proteins, such as Cdc6 and Myc. Remarkably, the HUWE1 protein showcases an aptitude for interacting with transcription factors, allowing it to assert dominance over their stability and transcriptional activity. This capacity plays a pivotal role in the modulation of diverse transcription factors, encompassing p53, Myc, and N-Myc.^139^ Yuan et al made a significant stride by associating diminished HUWE1 levels in gliomas with an unfavorable prognosis. The orchestrated HUWE1-mediated ubiquitination and subsequent degradation of N-Myc translated into the deactivation of the downstream DLL1-NOTCH1 signaling pathway, effectively impeding cell progression in glioblastoma multiforme.^139^ In another *in vitro* study on prostate cancer cells, Fan and colleagues illuminated the role of Jumonji C domain-containing 1A (a histone demethylase) in inhibiting HUWE1-induced c-Myc degradation. This regulatory interplay was pivotal in dictating the trajectory of prostate cancer development.^140^ The overexpression of HUWE1 leads to a decrease in the expression level of c-Myc, thereby inhibiting cell proliferation and migration.^141^ On the other hand, R1 (CDCA7L/RAM2/JPO2) hinders the transcription of HUWE1, consequently enhancing the stability of c-Myc in prostate cancer cells.^142^ These findings suggest that HUWE1 may act as a tumor suppressor in human prostate cancer.^140^

Understanding the Ub-mediated regulation of c-Myc in CSCs provides valuable insights into the intricate balance between self-renewal, differentiation, and survival. The involvement of diverse E3 ligases in governing c-Myc stability underscores the complexity of regulatory networks within CSCs. Unraveling these mechanisms not only enhances our comprehension of CSC biology but also opens avenues for developing targeted therapies aimed at disrupting c-Myc-driven tumorigenesis. Future research endeavors in this area promise to uncover additional layers of regulation, potentially offering novel strategies for precision medicine and improvement of cancer treatment outcomes.

## Ubiquitination of Nanog transcription factors in CSCs

The Nanog gene is situated on chromosome 12 of the human genome (and on chromosome 7 in mice), and it comprises multiple exons and introns that encode a protein consisting of 305 amino acids. Nanog is a highly expressed protein in ESCs and other stem cells, which plays a crucial regulatory role in maintaining the self-renewal and pluripotency of these cells.^143^ The expression level of Nanog is intricately linked to the state of stem cells; elevated levels of Nanog expression are crucial for maintaining stem cell self-renewal and pluripotency, while the absence or reduction of Nanog may result in stem cell differentiation and loss of pluripotency.^144^ Nanog exerts its influence on gene expression through interaction with other transcription factors, such as Oct4 and Sox2, as well as co-activator proteins, thereby forming a complex regulatory network. It plays a crucial role in the regulation of various pivotal genes, encompassing those associated with stem cell properties, cell cycle control, and determination of cellular fate.^145^^,^^146^ Moreover, Nanog has been implicated in tumorigenesis and progression due to its aberrant expression. Overexpression of Nanog has been observed in certain tumors, which is associated with the presence of CSCs as well as tumor invasion and metastasis.^147^

### The stability of Nanog protein is regulated by FBXW8

RACK1, a member of the Trp–Asp (WD) repeat protein family, is regarded as an adaptor protein that participates in multiple intracellular signaling pathways. Elevated levels of RACK1 mRNA or protein have been observed in clinical hepatocellular carcinoma samples by different research groups.^148^^,^^149^ RACK1 enhances Nanog expression in both hepatocellular carcinoma cells and mouse ESCs.^150^ Gao et al demonstrate that RACK1 directly binds to Nanog, thereby preventing the recruitment of E3 Ub ligase FBXW8 and subsequent Ub-dependent degradation.^151^ This process promotes self-renewal ability and chemoresistance in human liver CSCs while also maintaining mouse ESC function. Additionally, RACK1 modulates the nuclear translocation of Nanog, which is predominantly localized in the nucleus in mouse ESCs. These findings suggest that RACK1 facilitates the nuclear translocation of Nanog through direct binding to Nanog. With regards to Nanog transactivation, the knockdown of RACK1 significantly impeded the process in hepatocellular carcinoma HuH7 cells, while overexpression of RACK1 notably augmented it.^151^ A similar conclusion was reached by another study regarding the direct phosphorylation of Nanog by ERK1, which clearly identified Ser52 as the primary phosphorylation site and noted weak phosphorylation at Ser65 by ERK1.^152^ The phosphorylation of Nanog by ERK1 plays a crucial role in the degradation of Nanog via ubiquitination-mediated protein stability. This study elucidates the mechanism by which Nanog is degraded via FBXW8 as a recognition factor, with Nanog serving as a novel substrate for proteolytic processing through Ub-mediated degradation.^152^

### The stability of Nanog protein is regulated by SPOP

SPOP is a constituent of the Cullin 3-RING E3 Ub ligase complex, which participates in various biological processes, including protein degradation, transcriptional regulation, DNA damage repair, and cell cycle control through its interactions with other proteins.^134^ The fundamental architecture of SPOP comprises BTB and POZ domains at the N-terminus and a MATH domain at the C-terminus. The BTB/POZ domains exhibit the ability to engage in protein–protein interactions and can establish complexes with other proteins. The MATH domain plays a crucial role in mediating the interaction between SPOP and its substrate through binding and recognition.^153^ It has been demonstrated that SPOP acts as a tumor suppressor in prostate cancer by primarily targeting the pluripotency-maintaining transcription factor Nanog for polyubiquitination and subsequent degradation, thereby inhibiting prostate CSC traits. Notably, SPOP mutants derived from prostate cancer were found to cluster in their substrate-recruited MATH structural domains, thereby disrupting SPOP's ability to bind to and promote Nanog for polyubiquitination and degradation, ultimately suppressing the characterization of prostate CSCs. These findings suggest that SPOP mutations may contribute to tumorigenesis by up-regulating the pluripotency factor Nanog. This up-regulation, in turn, confers the characteristic features of CSCs.^154^ Another insightful study demonstrated that SPOP impedes tumor progression in testicular germ cells by facilitating the degradation of developmental pluripotency-associated 2 and reducing the levels of Nanog and Oct4.^155^ In line with these findings, SPOP restricts the stability and function of NANOG to impede the progression of prostate cancer. The promotion of ubiquitination and subsequent UPS-dependent degradation of Nanog is facilitated by SPOP, which relies on the interaction between Nanog-SPOP involving the MATH structural domain of SPOP and the SPOP-binding consensus degron of NANOG.^154^^,^^156^

While the current understanding of Nanog ubiquitination in CSCs provides valuable insights, future investigations should delve deeper into the crosstalk between Nanog and other Ub ligases. Unraveling additional layers of regulation may reveal new players in the dynamic control of Nanog stability and function in CSCs. Moreover, deciphering how these regulatory networks integrate with signaling pathways and epigenetic modifications will enhance our ability to target CSCs effectively. The development of therapeutic strategies aimed at disrupting Nanog-associated tumorigenesis holds immense promise, and ongoing research in this field is poised to bring about transformative advancements in cancer treatment.

## Ubiquitination of KLF4 transcription factors in CSCs

KLF4, a member of the Krüppel-like factor family, functions as a transcription factor. Its dynamic functionality encompasses both activation and repression domains, facilitating the intricate orchestration of coactivator and corepressor recruitment. Additionally, KLF4 showcases the presence of three zinc fingers, each adept at specifically binding to guanine-cytosine-rich sequences, exemplified by CACCC motifs commonly encountered in gene regulatory promoters and enhancers.^157^^,^^158^ It plays a crucial regulatory role in various biological processes and is essential for maintaining stem cell properties, regulating differentiation, drug resistance, as well as tumorigenesis and development. KLF4 plays a pivotal role in maintaining stem cell properties in CSCs by regulating the expression of multiple genes related to stemness, thereby enabling self-renewal and proliferation.^159^ Following the seminal discovery of KLF4's role in somatic cell reprogramming to induced pluripotent stem cells, extensive research has been dedicated to exploring KLF4's influence, particularly in the context of CSCs. As a transcription factor, KLF4 assumes a multifaceted role in the intricate landscape of post-translational modifications. It is intricately involved in the mechanistic regulation of various post-translational modifications, including acetylation, SUMOylation, phosphorylation, and ubiquitination.^160^, ^161^, ^162^, ^163^ Multiple E3 ligases, such as VHL, Fbox protein 32, and β-TrCP, have been identified as targets for KLF4 ubiquitination and degradation in numerous studies.^164^^,^^165^

### The stability of KLF4 protein is regulated by β-TrCP

Through controlled expression of the transcription factor KLF4, along with other factors, adult fibroblasts can be reprogrammed into induced pluripotent stem cells that exhibit similar behavior to ESCs.^166^ KLFs are a subset of Cys2-His2 zinc finger proteins that function as DNA-binding transcription factors.^166^ Three members of this family, namely KLF2, KLF4, and KLF5, exhibit high expression levels in undifferentiated mouse ESCs but are rapidly down-regulated during the early stages of differentiation.^166^ KLF4 is ubiquitously expressed in various tissues and participates in numerous physiological processes, including the regulation of self-renewal of ESCs.^167^ An illuminating study delved into the intricate mechanisms at play, revealing that either ERK1 or ERK2 binds to the activation domain of the KLF4 C-terminal region. In mouse ESCs, this binding prompts the direct phosphorylation of Ser123 within KLF4. However, this phosphorylation event does not go unnoticed — it emerges as a pivotal determinant dictating the transcriptional activity of KLF4. This consequential phosphorylation, occurring at Ser123, precipitates the differentiation of mouse ESCs.^168^ Interestingly, the converse scenario unfolds when ERK-mediated inhibition intervenes. In such instances, ERK inhibition of KLF4 phosphorylation bolsters KLF functional activity, resulting in the obstruction of ESC differentiation. Notably, phosphorylation of KLF Ser123 by ERK1 or ERK2 induces the recruitment and binding of β-Trcp2, a component of the Ub E3 ligase, to the N-terminal domain (residues 1–140) of KLF4. Moreover, the binding of β-Trcp leads to KLF4 ubiquitination and subsequent degradation via the proteasomal pathway.^168^ In summary, these revelations weave a coherent molecular foundation for KLF4's direct participation in the orchestration of self-renewal within ESCs and CSCs.

### The stability of KLF4 protein is regulated by VHL

The VHL gene is a gene located in the 3p25-26 region of the human chromosome and consists of three exons. The promoter region of the VHL gene is located upstream of exon 1 and contains important sequence elements that regulate gene transcription.^169^ In addition, the VHL gene has some important regulatory sequences, such as transcription factor binding sites and splicing sites. Mutations in the VHL gene have been linked to a range of tumors, including clear-cell renal carcinoma, retinal and cerebellar hemangioblastoma, as well as pheochromocytoma.^170^ The VHL protein serves as the substrate recognition component of the CBC VHL E3 Ub ligase complex, which is composed of pVHL, elongins B and C, Cullin 2, and RING-H2 finger protein RBX1. CBC VHL binds to and degrades the alpha subunits of heterodimeric hypoxia-inducible transcription factors HIF1 and HIF2. Growing evidence suggests that the activity of pVHL extends beyond the regulation of HIF, encompassing other substrates for its Ub ligase function such as atypical protein kinase Cλ, pVHL-interacting DUBs (pVHL-interacting deubiquitinating enzyme 1/2), and two subunits of RNA polymerase II (Rpb1 and Rpb7).^170^ Gamper et al unearths the transcription factor KLF4 as a direct target of pVHL's E3 ligase activity, establishing this connection within both mouse and human cells. The study provides compelling evidence bolstering the role of pVHL-mediated degradation of KLF4 in VHL-dependent growth control.^171^ This notion is further substantiated by the observation that replenishing pVHL triggers cell cycle arrest due to the cumulative buildup of KLF4 and the subsequent upregulation of p21 within colon cancer cells. Intriguingly, a reverse scenario unfolds in colon cancer tissues, where KLF4 levels are diminished while pVHL expression surges.^171^ ITGB4 is an oncogene that exhibits overexpression, which is correlated with aggressive phenotypes and unfavorable prognoses in breast, lung, pancreatic, cervical, and gastric cancers.^172^ In glioma, KLF4 directly binds to the ITGB4 promoter, promoting its transcription and contributing to increased expression of ITGB4 in glioma. In a remarkable feedback loop, ITGB4 interacted with KLF4 and reduced its binding to E3 ligase VHL in glioma cells, thereby enhancing KLF4 stability and increasing KLF4 expression. Collectively, these studies unveil a novel feedback loop linking KLF4 and ITGB4 that contributes to the self-renewal of glioma stem cells and the development of gliomas. In essence, these studies together usher in a deeper understanding of the intricate mechanisms governing pivotal characteristics in stem cells. This newfound insight dances on the delicate equilibrium between self-renewal and differentiation, fostering advancements in comprehending both embryogenesis and the complex realm of cancer biology. Moreover, this enhanced understanding holds the potential to serve as a valuable resource for cell transplantation, allowing the replenishment of cells lost due to injuries or diseases.

Understanding the Ub-mediated regulation of KLF4 in CSCs not only sheds light on the delicate equilibrium between self-renewal and differentiation but also unveils potential therapeutic targets for cancer treatment. The intricate interplay between KLF4, E3 ligases like β-TrCP and VHL, and other cellular factors orchestrates a molecular symphony governing stem cell property. As research advances, uncovering additional players in the regulatory network and deciphering the crosstalk with signaling pathways will refine our comprehension of KLF4's role in both embryogenesis and cancer biology. Targeting KLF4 ubiquitination pathways holds promise for developing strategies to manipulate CSC characteristics and advance cancer therapies. This burgeoning field opens avenues for innovative interventions that harness the inherent regulatory mechanisms within CSCs, bringing us closer to effective treatments for a range of cancers.

Taken together, the molecular mechanisms by which E3 Ub ligases target CSC-associated transcription factors (SOX2, OCT4, KLF4, and Myc) are diverse, including regulation of their stability, function, and subcellular localization. These studies provide an important molecular mechanistic basis for a deeper understanding of stem cell self-renewal and CSC formation and maintenance.

## Deubiquitinating enzymes and CSCs

DUBs are a class of enzymes capable of catalyzing the deubiquitylation process. In human cells, there exist approximately 100 DUBs, with the largest subgroup being USPs that encompass around 60 members.^173^ Other groups comprise Ub carboxy-terminal hydrolases, OTU, Machado-Joseph disease proteases, JAB1/MPN/MOV34 proteases, monocyte chemoattractant protein-inducible proteins, and a novel motif that interacts with the MIU-containing DUB family. All DUBs are cysteine proteases, with the exception of AMM which functions as a Zn^2+^ metalloprotease. The primary biological role of DUBs is to catalyze the removal of Ub chains from proteins, thereby stabilizing protein structures and preventing proteasomal degradation. Additionally, DUBs are involved in the processing and maturation of Ub precursors, as well as the recycling and editing of Ub chains.^174^ The UPS is regulated by approximately 600 E3 Ub ligases, with relatively few DUBs playing a crucial role in maintaining balance. This highlights the significance of DUBs in both normal and cancer cells. Recently, multiple studies have demonstrated that CSC-associated proteins regulate the fate of CSCs through the Ub proteasome system, including but not limited to SIRT1, p53, PTEN, LSD1, and PRCs.^175^^,^^176^

### PTEN

PTEN, initially discovered in 1997, is among the most frequently mutated tumor suppressor genes in human cancer.^177^ The deletion or inactivation of PTEN has been closely linked to the development and progression of various types of tumors.^178^ Additionally, PTEN functions as a negative regulator of the PI3K/AKT cascade, which is responsible for controlling cell growth, proliferation, survival, and metabolism.^179^ Furthermore, PTEN deficiency leads to the formation of CSCs with self-renewal and multilineage differentiation capabilities.^180^ ATXN3 functions as a transcriptional repressor by inhibiting PTEN transcription, while maintaining PTEN protein stability.^181^ Conversely, USP18 overexpression stabilizes PTEN proteins, and USP18 inhibition primarily reduces cytoplasmic PTEN.^182^ Another study demonstrated that BCR-ABL promotes the exclusion of PTEN from the nucleus in chronic myeloid leukemia. BCR-ABL directly regulates PTEN partitioning through interaction with HAUSP, which deubiquitiates PTEN. Furthermore, BCR-ABL modulates HAUSP via direct tyrosine phosphorylation of multiple residues. The exclusion of PTEN from the nucleus may confer a proliferative advantage and abnormal expansion to hematopoietic progenitor cells in chronic myeloid leukemia.^183^ Therefore, these findings suggest that the PML/HAUSP/PTEN pathway plays a critical and druggable role in the pathogenesis of chronic myeloid leukemia.

### PRCs

PRCs are crucial epigenetic regulatory complexes responsible for chromatin remodeling by forming large polyribonucleic acid complexes and inducing gene silencing through specific histone modifications.^184^ In CSCs, the aberrant functioning of PRCs may result in abnormal self-renewal and dysregulated differentiation of CSCs, thereby facilitating the formation and progression of tumors.^185^ PRC1 (including BMI-1, RING1a, and RING1b/RNF2) and PRC2 (including EZH2, EED, and SUV12) are typically classified into two groups. The subsequent histone modifications induced by these complexes play a crucial role in maintaining stable silencing of ectochromatin and surface heterochromatin 186. PRC2 mediates transcriptional repression by catalyzing the trimethylation of histone H3 at lysine 27, while PRC1 induces chromatin compaction through its mono-ubiquitination of histone H2A at lysine 119 (H2AK119).^187^ For instance, FBXL10 is a constituent of the atypical PRC1 complex, which serves as the primary E3 ligase for H2A mono-ubiquitination in mouse ESCs. FBXL10 interacts with Ring1B and nervous system polycomb 1 to form the atypical PRC1 complex that is essential for the mono-ubiquitination of H2AK119 in mouse ESCs.^188^ Moreover, the promotion of H2A mono-ubiquitination by FBXL10 is contingent upon the binding region of nervous system polycomb 1 located in its C-terminal. Genetic analyses have demonstrated that FBXL10's DNA-binding ability and integration into PRC1 are essential for H2AK119 ubiquitination. The findings suggest that FBXL10 plays a pivotal role in the regulation of Ring1B recruitment to its target genes and H2AK119 ubiquitination in ESCs.^188^ The silencing of polycomb genes may necessitate the ubiquitination of H2A in PRC1 and the deubiquitination of H2A by the polycomb repressive deubiquitinating enzyme.^189^ In certain types of cancer, PRC1 can undergo deubiquitination by USP7, USP11, and USP26.^190^ Overall, further investigation is required to fully understand the mechanism by which PRC regulates DUBs.

### LSD1

In CSCs, research has indicated that LSD1 plays a role in the regulatory mechanism of the UPS. The regulatory mechanism plays a crucial role in preserving the stemness of CSCs and promoting tumor progression.^191^, ^192^, ^193^ In gliomas, USP7, a deubiquitinating enzyme, stabilizes LSD1 to facilitate the genesis and metastasis of glioblastoma cells by suppressing the p53 signaling pathway.^194^ Moreover, the up-regulation of LSD1 was observed in multiple cancer cell lines and breast tumor samples with the overexpression of USP28. USP28 functions as a bona fide deubiquitinating enzyme for LSD1, as it interacts with and stabilizes LSD1 through the process of deubiquitination. Knockdown of USP28 results in the destabilization of LSD1, leading to suppression of CSC-like features *in vitro* and tumorigenicity *in vivo*.^195^

### SIRT1

SIRT1, a member of the sirtuin family, is an NAD^+^-dependent histone deacetylase that modulates the activity of various intracellular proteins, including p53.^196^ SIRT1 governs a multitude of cellular processes, encompassing senescence, DNA repair, cell cycle progression, homeostasis maintenance, and cellular aging.^197^ SIRT1 plays a crucial role in the maintenance of self-renewal and differentiation processes in both mouse ESCs and hematopoietic stem cells.^198^ Ling Li et al discovered that acute myeloid leukemia stem/progenitor cells display varying degrees of SIRT1 overexpression, and targeting leukemic stem cells through inhibition of SIRT1 represents a promising approach.^199^ Furthermore, a separate study demonstrated that USP22, a Ub-specific peptidase, acts as a positive regulator of SIRT1 by interacting with and stabilizing the protein through the removal of polyubiquitin chains. In contrast, the knockdown of endogenous USP22 results in the destabilization of SIRT1, inhibition of SIRT1-mediated p53 deacetylation, and enhancement of p53-dependent apoptosis. Moreover, the genetic elimination of the USP22 gene results in the destabilization of SIRT1, increased transcriptional activity of p53, and premature embryonic demise in mice.^200^

### p53

p53, a widely recognized tumor suppressor, is renowned for its pivotal role as the “guardian of the genome”, and impaired p53 expression has been observed in nearly all human cancers. Additionally, p53 is recognized as a pivotal regulator of stem cell properties, governing the differentiation of embryonic and adult stem cells, impeding the reprogramming of induced pluripotent stem cells, and suppressing cancer stemness, which underlies drug-resistant tumorigenesis.^201^ The literature indicates that HAUSP, a USP, has been demonstrated to deubiquitinate p53 and that up-regulation of HAUSP results in the stabilization of p53.^202^ However, depletion of HAUSP in cells did not result in the expected decrease of p53 levels. Instead, it led to an increase in p53 levels due to the ability of HAUSP to bind to and deubiquitinate MDM2. In fact, HAUSP appears to have a stronger affinity for binding to MDM2 than to p53.^203^ USP10 has been reported to act as a deubiquitinase for p53, thereby counteracting MDM2-mediated nuclear export and degradation of p53.^204^ Moreover, ataxin-3, the deubiquitinase associated with Machado-Joseph disease, interacts with p53 and functions as a novel DUB for p53.^205^ Additionally, the OTUD subfamily comprises OTUD5 and OTUD1, both of which have been reported to play a role in stabilizing p53.^206^ The stability of p53 in response to DNA damage or genotoxic stress is regulated by OTUD5, while cell growth and apoptosis are regulated by OTUD1 by increasing the stability and activity of p53.^207^ OTUD1 shares similarities with USP10 and OTUB1. USP10 regulates cell growth and apoptosis by controlling the stability and activity of p53 in response to cellular stresses, such as DNA damage.^204^ In summary, the primary function of these DUBs is to regulate tumorigenesis and intervene by modulating the stability and activity of stem cell factors through deubiquitination (Table 1). DUBs emerge as master conductors in the intricate symphony of CSC regulation, presenting promising avenues for targeted therapeutics. Decoding the specific interactions and mechanisms of these enzymes holds the key to unraveling the complexities of CSC regulatory networks and advancing innovative strategies in cancer treatment.

**Table 1:** Deubiquitinating enzyme-associated stem cell factors regulate tumorigenesis through deubiquitination regulation.

Stemness-related factors	Deubiquitinating enzymes	Function	Cancer-related types	References
ID proteins	USP1	Protein stabilization	Leukemia, osteosarcoma, breast cancer	208, 209, 210
PTEN	ATXN3, USP18, USP7	Transcriptional regulation, protein stabilization and location, tumor suppression	Prostate cancer, lung cancer, and endometrial cancer	181,182,211
p53	USP2a, Ataxin-3, USP7, OTUB1, OTUD1, OTUD5, USP11, USP10	Protein stabilization	Lung cancer, gastric cancer, pancreatic cancer, ovarian cancer	206,207,212, 213, 214, 215, 216
SIRT1	USP22	USP22 functions as a novel regulator of the SIRT1-STAT3 signaling pathway	Colorectal cancer	217
Sox2	USP22, USP9X	Controls the transcriptional activity and proliferation of malignant cells	Salivary adenoid cystic carcinoma, lung cancer, brain cancer, prostate cancer, pancreatic cancer	218, 219, 220
PRC1	USP7, USP11, USP26	Stabilization of the PRC1 complex	Prostate cancer, ovarian cancer	190
PRC2	BAP1	Regulation of gene expression	Prostate cancer	221
C-myc	USP37,^18^ USP22,^19^ USP36,^20^ USP28^21^	Protein stabilization	Lung cancer, breast cancer	222,223
Nanog	USP21, USP3, USP16	Protein stabilization, stem cell pluripotency	Mouse embryonic stem cell, glioma, ovarian cancer	224,225
Oct4	USP7, USP44	Protein stabilization	Female germline stem cell tumors, testicular germ cell tumors, breast cancer	225,226
KLF4	USP10, DUB3, ATXN3,	Protein stabilization, enhancement of chemotaxis in hepatocellular carcinoma, enhanced the metastasis of breast cancer cells	Breast cancer, lung cancer, hepatocellular carcinoma	227, 228, 229
Sirt1	USP22	Protein stabilization, acetylation,	Colorectal cancer, breast cancer	230,231
REST	USP7, USP15	Protein stabilization	Glioma	232,233

## DUBs and CSC microenvironment

Numerous studies have demonstrated the pivotal role of the CSC microenvironment in sustaining their stemness. The microenvironment specific to tumors encompasses stromal and immune cells, cytokine and growth factor networks, hypoxic regions, as well as the extracellular matrix. In this review, we will discuss the regulatory mechanisms of the CSC microenvironment mediated by ubiquitination in terms of both hypoxia and inflammation.^234^^,^^235^

### Hypoxia

The unlimited capacity for self-renewal in cancer cells leads to an increased population of epigenetically heterogeneous cells and tumor volume, consequently resulting in a reduction of oxygen concentration in the surrounding environment. Hypoxia is widely recognized as a prominent characteristic of the tumor microenvironment and a potential determinant of the CSC phenotype. The HIFs, such as HIF-1α and HIF-2α, are prototypical transcription factors that are strongly activated in malignant tumors under hypoxic conditions.^236^ Their cytological functions encompass the maintenance of stemness and the facilitation of cellular proliferation. Additionally, HIFs stimulate angiogenesis and metastasis while also promoting cancer cell survival. For instance, in hypoxic regions, cancer-associated fibroblasts express CD44 on the surface of CSCs at high levels, which facilitates metastasis and contributes to stemness maintenance.^237^^,^^238^ For example, in the presence of oxygen, VHL tumor suppressor proteins interact with HIF proteins, thereby facilitating ubiquitination and subsequent degradation of HIF proteins. This process effectively maintains low levels of expression for these stemness-associated factors.^239^ Moreover, the stability of HIF protein can be regulated by DUBs such as USP8, USP19, and USP28.^240^, ^241^, ^242^ Additionally, USP52 plays a crucial role in the P-body complex to prevent degradation of HIF-1α mRNA.^243^

### Inflammation

Inflammatory processes and CSCs are being increasingly acknowledged as significant contributors to the development of tumors. Emerging evidence suggests that CSCs are implicated in key cancer hallmarks, including metastasis, drug resistance, and disease recurrence. However, the precise interplay between CSCs and inflammation remains to be elucidated. The discovery of DUB3 as a novel DUB for Slug and Twist was made. Overexpression of DUB3 exhibited a dose-dependent increase in the protein levels of Slug and Twist, whereas knockdown of DUB3 resulted in a decrease in their protein levels. Significantly, DUB3 interacts with Slug and Twist to inhibit their degradation, thereby enhancing the migratory, invasive, and CSC-like properties of breast cancer cells. Interestingly, DUB3 has been identified as an early response gene that is up-regulated upon exposure to inflammatory cytokines such as IL-6, which plays a pivotal role in the growth and metastasis of breast cancer cells, as well as in the maintenance of breast CSCs. Moreover, DUB3 plays a crucial role in IL-6-induced EMT by stabilizing Slug and Twist.^244^ Additionally, Vera Levina and colleagues demonstrated an elevation in the levels of IL-6 and granulocyte colony-stimulating factor within lung CSCs.^245^ Furthermore, the inhibition of IL-8, a chemokine that regulates the properties of CSCs in renal cell carcinoma, was found to be associated with decreased levels of the deubiquitinating enzyme USP21. Mechanistically, USP21 binds to the promoter region of IL-8 and facilitates transcription initiation. These findings suggest that targeting the USP21/IL-8 axis may hold promise as a therapeutic strategy for renal cell carcinoma.^246^

Thus, beyond their role within cancer cells, DUBs exert a profound influence on the CSC microenvironment. The intricate interplay unfolds in the two distinct dimensions (hypoxia and inflammation), each of which sculpts the destiny of CSCs. Unraveling these dimensions not only expands our understanding of CSC regulation but also illuminates potential therapeutic avenues. Targeting DUB-mediated pathways within the CSC microenvironment presents a nuanced strategy for disrupting the intricate web of hypoxia and inflammation, offering novel prospects in cancer therapeutics.

## The signaling network connecting CSCs and ubiquitination

The development of cancer is attributed to aberrant cell proliferation caused by genetic mutations. While CSCs, similar to other cancer cells, originate from gene-driven mutations, they possess distinct developmental characteristics compared with non-stem cells, including disparities in epigenetic modifications and gene expression profiles.^19^ The involvement of the Ub proteasome in numerous alterations to CSC signaling pathways provides a preliminary justification for the development of compounds that target CSCs. Signaling pathways that govern the self-renewal and differentiation of CSCs encompass a range of extensively researched factors, including Wnt, Hh, Notch, and Hippo.^247^^,^^248^

### Ubiquitination in the Wnt/β-catenin signaling pathway

The Wnt/β-catenin signaling pathway plays a crucial role in tissue regeneration and cellular fate determination and is intricately involved in tumorigenesis. The Wnt signaling pathway is highly complex and evolutionarily conserved, encompassing over a dozen Wnt ligands along with their corresponding receptors. It can be classified as both a β-catenin-dependent pathway and an atypical Wnt pathway.^249^^,^^250^ The Wnt signaling pathway plays a crucial role in embryonic development, homeostasis, cell differentiation, polarity, proliferation, and migration by binding Wnt ligands to co-receptors for coiled-coil proteins and low-density lipoprotein receptor-associated proteins (LRPs), which initiate the Wnt/β-catenin signaling cascade. This ultimately leads to β-catenin stabilization, nuclear translocation, and activation of target genes.^251^ Various signaling factors facilitate the up-or down-regulation of Wnt signaling via post-translational modifications, and dysregulated regulation has been linked to several human malignancies. Among the numerous post-translational modifications involved, ubiquitination and deubiquitination play crucial roles in regulating most Wnt signaling factors. Ubiquitination, mediated by E3 ligases, attaches Ub molecules to target proteins and typically triggers proteasomal degradation of key components in the Wnt signaling pathway, including β-catenin, Axin, GSK3, and Dvl.^252^ In contrast, deubiquitination mediated by DUBs facilitates the segregation of Ub and modulates the stability of signaling factors. In the Wnt signaling pathway, the UPS plays a crucial role in regulating the stability of β-catenin, a key signaling factor that triggers the expression of Wnt target genes.

The SCF complex mediates the attachment of polyubiquitin chains to K19 and K49 residues on β-catenin by specifically binding to its substrate recognition motif.^253^ Moreover, β-Trcp, a cyclic E3 ligase, serves as a representative E3 ligase responsible for targeting β-catenin.^254^ Siah1, in conjunction with ubiquitin-conjugating enzyme Ha (an E2 enzyme), acts as an E3 ligase for β-catenin and promotes its ubiquitination at K666 and K671. It has been shown that Siah-1 induces ubiquitination of membrane-bound β-catenin irrespective of p53 activation. The ubiquitination of cytoplasmic β-catenin by Siah-1 is induced through high expression of Siah-1 upon activation of p53. Unlike β-Trcp, p53 can induce ubiquitination and degradation of β-catenin regardless of its phosphorylation status.^255^^,^^256^ Furthermore, Ozz-E3 ligase mediates ubiquitination of sarcoplasmic-associated β-catenin, while Jade-1 targets nuclear β-catenin for degradation through ubiquitination. Conversely, EDD/Rad6B/FANCL-mediated ubiquitination enhances the stability of β-catenin. Specifically, EDD links K29- and K11-linked polyubiquitin chains whereas Rad6B connects K63-linked polyubiquitin chains.^257^

Recent research has emphasized the role of Wnt activation in regulating CSCs across various cancer types, including colorectal, breast, skin, nervous system, intestinal, lung, urinary tract, and blood cancer.^258^^,^^259^ Maintaining the delicate equilibrium between ubiquitination and deubiquitination processes is essential to uphold normal cellular activities, particularly within the intricate world of Wnt and its receptor Frizzled. Within this dynamic landscape, an interplay of elements that holds the key to cellular fate emerges. An illustrative example presents itself with the interaction of USP2a and β-catenin. This intricate alliance stabilizes and simultaneously propels β-catenin towards ubiquitination, ultimately ushering it into an enhanced state of nuclear accumulation and transcriptional prowess. The orchestration culminates in the up-regulation of Wnt/β-catenin target genes, setting a cascade of cellular events in motion. In a quest to tap into the therapeutic potential of this regulatory axis, the small molecule inhibitor ML364 steps into the scene, effectively curtailing β-catenin in cancer cells by quelling the activity of USP2a. This novel insight suggests that targeting USP2 could potentially hold the key to reining in the oncogenic protein β-catenin 260. Additionally, Jiang et al discovered that the transmembrane E3 Ub ligases ZNRF3 and RNF43 play a crucial role in promoting the degradation of Wnt receptors, thereby functioning as tumor suppressors.^261^ RNF43/ZNRF3 plays a crucial role in regulating the Wnt signaling pathway by controlling the ubiquitination-mediated stability of the Frizzled receptor, and mutations in RNF43/ZNRF3 lead to typical activation of Wnt signaling by maintaining β-catenin stability. These mutations are frequently observed in various cancers such as pancreatic, adrenal, and gastric cancers. Furthermore, ZNRF3 and RNF43 facilitate the interaction between the signaling protein DVL and the Wnt receptor, unveiling an unexpected dual functionality of DVL within Wnt signaling. The DVL protein functions as a positive regulator of Wnt signaling by transducing signals induced by Wnt proteins, which are involved in both canonical and non-canonical pathways, thereby enhancing the stability of β-catenin. Consequently, a multitude of cancers exhibit DVL overexpression.^262^ For instance, abnormal spindle microcephaly associated enhances β-catenin stability by binding to DVL in prostate cancer.^263^ Additionally, the binding of the axin complex to DVL triggers the phosphorylation of LRP5/6 at the PPPSP motif, leading to the recruitment of more axin complexes to the cell membrane. The translocation of axin complexes to the cellular membrane does not lead to Ub-mediated degradation of β-catenin; instead, this non-degraded β-catenin translocates to the nucleus and interacts with TCF/LEF, thereby initiating the transcriptional activation of Wnt target genes.^264^^,^^265^

As an E3 ligase, β-Trcp plays a vital role in the ubiquitination of β-catenin, which acts as a negative regulator for Wnt target gene transcription. Mutations in β-Trcp lead to increased expression of Wnt target genes and disruption of β-catenin ubiquitination. β-Trcp ubiquitinates a diverse range of proteins, including β-catenin, and modulates the activity of discs large homolog 5, which suppresses the proliferation of hepatocellular carcinoma cells, as well as AE-binding protein 2, which promotes proliferation and confers cisplatin resistance in ovarian cancer cells.^266^^,^^267^ Additionally, β-Trcp regulates the stability of two oncogenes in pancreatic and lung cancer, namely YAP1 and max interactor 1 respectively.^268^ This is achieved through ubiquitination and proteasomal degradation, which inhibits c-Myc transcriptional activity in lung cancer.^269^ A separate study demonstrated that Wnt acts as a bridge between Frizzled-LRP5/6 proteins and phosphorylates LRP5/6 via casein kinase 1α. This subsequently recruits a “destructive complex” to the cell membrane, inhibits β-catenin's phosphorylation and ubiquitination, and promotes its accumulation in the nucleus, thereby regulating CSC.^270^^,^^271^

Furthermore, the TCF family competitively interacts with the β-catenin and TLE families in nuclear processes directly associated with Wnt target gene expression.^252^^,^^272^ Operating as a critical steward, TCF adopts a dual role, depending on the presence or absence of Wnt proteins. In the absence of Wnt cues, TCF takes on the mantle of a negative regulator, steering the TLE family binding via ubiquitination-mediated degradation of cytoplasmic β-catenin. However, with the entrance of Wnt proteins, β-catenin assumes a new identity, shuttling into the nucleus and partnering with TCF in orchestrating the symphony of transcription for Wnt target genes. Into this narrative steps TCF/LEF1, a substrate subject to the hand of the RING-type E3 ligase Neurodap1 (Pja2, Praja Ring Finger ubiquitin ligase 2), serving as the harbinger of ubiquitination-induced degradation of TCF/LEF1 protein levels.^273^^,^^274^ This process is implicated in the regulation of self-renewal and differentiation in ESCs. Another character in this story is EDD, the E3 ligase of β-catenin, orchestrating the choreography of Ub-mediated degradation of the TCF/LEF family. Nemo-like kinase-associated ring finger protein enters the scene and weaves Ub into the tale through the E2 Ub ligase E2-25K, a vital component in the inhibition of axial axis formation in Xenopus embryos.^275^ The theme of ubiquitination is woven throughout the Wnt narrative, extending its influence over various players within the pathway. For instance, the partnership of USP2a and β-catenin is a dynamic duo that elevates β-catenin to nuclear prominence, augmenting its transcriptional prowess. Through a complex ballet of interactions, USP2a binds to the N-terminal armadillo repeat motif domain or C-terminal domain of β-catenin, ushering in an expression surge of Wnt/β-catenin target genes.^260^ Meanwhile, USP4 emerges as a specific DUB for β-catenin, steering its expression and orchestrating β-catenin-mediated transcription. With its C-terminus hosting the catalytic domain responsible for β-catenin binding, USP4 propels this dance toward the nucleus. In the realm of colon cancer, this symphony takes on a deeper resonance as up-regulated USP4 and β-catenin herald the crescendo in colon cancer tissues. The removal of USP4 from the equation triggers a harmonious reduction in the invasion and migration abilities of colon cancer cells, showing USP4's potential as a target for future anticancer therapies.^276^ Yet another player, USP6, enters the narrative, casting its influence over the Wnt/β-catenin signaling pathway. Its deubiquitinating prowess shines as it engages with Frizzled, shifting the balance of Frizzled levels. This intricate play of ubiquitination and deubiquitination underpins a complex interplay between players that ultimately impacts Wnt signaling.^277^

**Figure 5:**
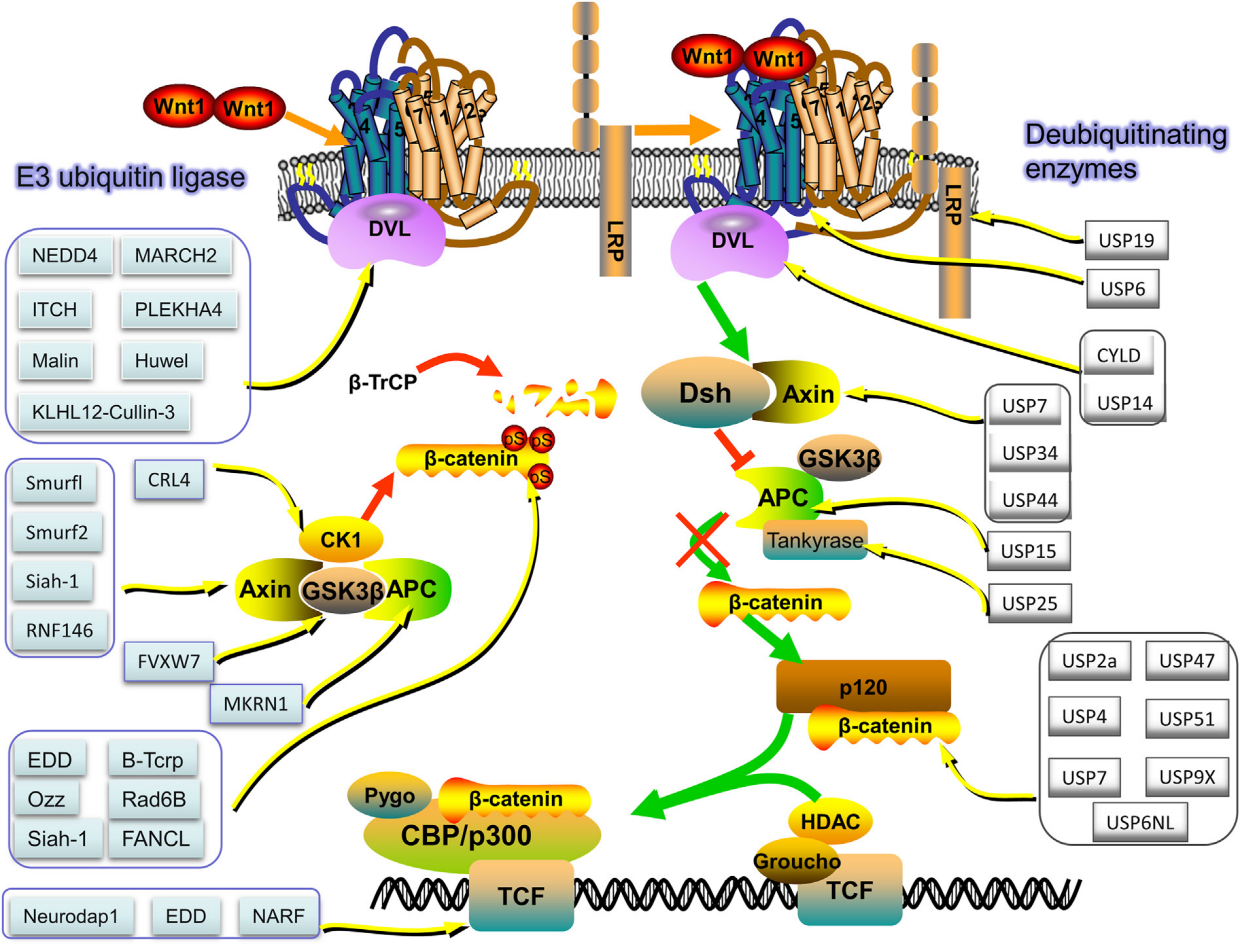
Wnt signaling-associated ubiquitination and deubiquitination.

USP7, a dual modulator of the Wnt signaling pathway, exerts both positive and negative effects. Specifically, USP7 impedes Wnt/β-catenin signaling by enhancing axin stability and reducing the expression of β-catenin and its downstream targets.^278^ The deubiquitination of BCL9 by USP9X facilitates the assembly of the β-catenin-BCL9-PYGO complex, thereby augmenting the transcriptional activity of Wnt/β-catenin target genes. Moreover, USP9X-mediated deubiquitination of BCL9 enhances breast cancer cell proliferation and invasion, implicating its involvement in Wnt/β-catenin signaling and breast carcinogenesis.^279^ Yet, the tapestry of Wnt signaling extends further with USP14, known for its role in the deubiquitination of Dvl. Inhibition of USP14 sets off a cascade, culminating in heightened polyubiquitination of DVL, significantly hampering the downstream effects of Wnt signaling.^280^ On a divergent note, USP15, a guardian of stability, intervenes by stabilizing APC, consequently bolstering the stability of CSN, CRL, and the β-catenin destruction complex.^281^ Collectively, these numerous E3 ligases and DUBs are associated with the regulation of the Wnt signaling pathway (Fig. 5), which plays a crucial role in cell proliferation, migration, stem cell pluripotency regulation, self-renewal, and differentiation. Therefore, abnormal activation of the Wnt pathway is believed to promote CSC progression and tumorigenesis.

### Ubiquitination in the Notch signaling pathway

**Figure 6:**
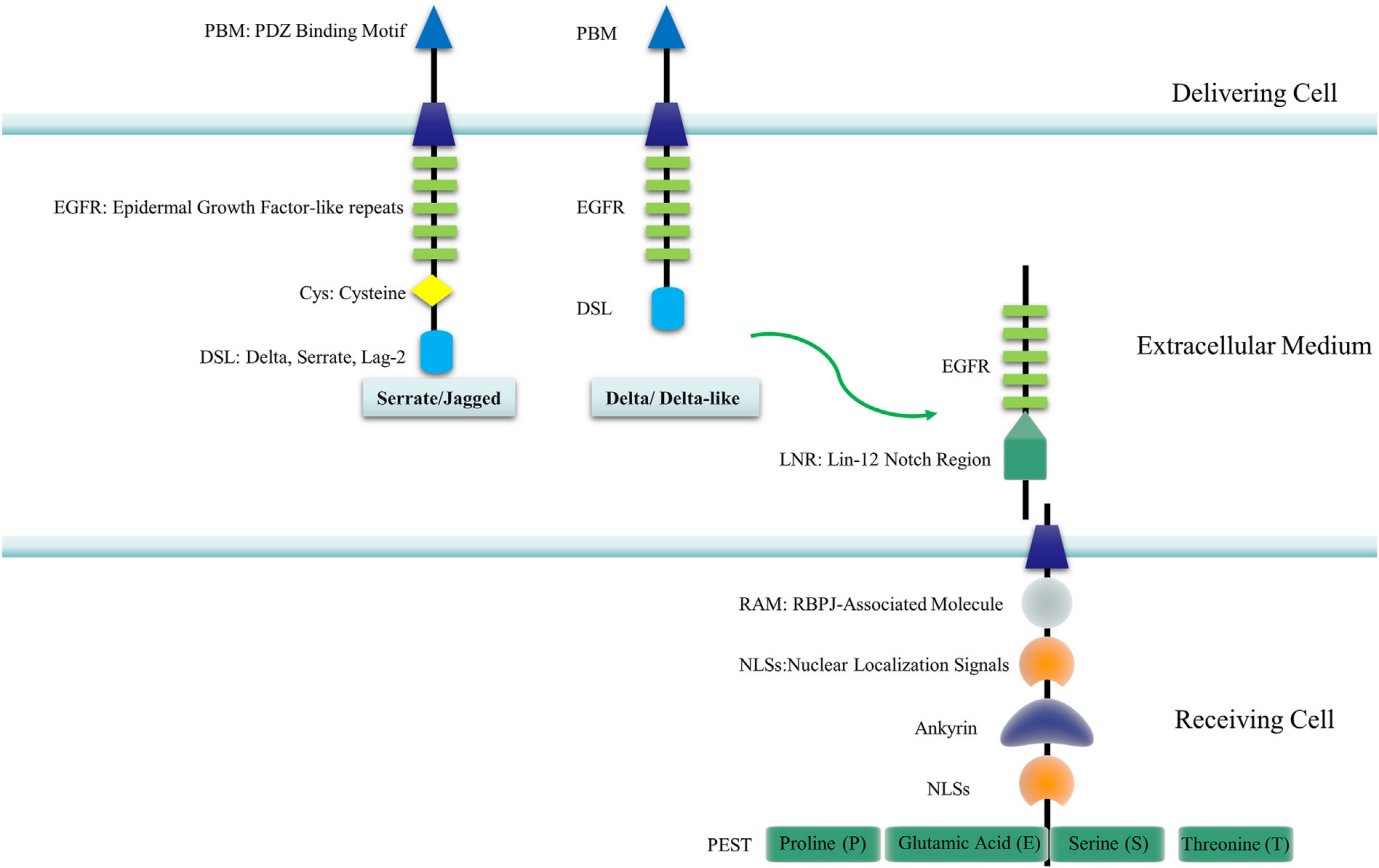
Structure of Notch receptor and DSL (Delta, Serrate, Lag2) ligands.

The Notch signaling pathway is an evolutionarily conserved pathway that plays a crucial role in various aspects of tumor biology, including the progression of CSCs, angiogenesis, and tumor immunity. The Notch pathway primarily consists of Notch receptors (Notch 1–4) and Notch ligands (Jagged 1, Jagged 2, delta-like ligand-1/3/4) (Fig. 6). Upon ligand binding to the receptor, the Notch intracellular domain is released into the nucleus via a three-step cleavage process facilitated by γ-secretase.^282^ This subsequently triggers the activation of transcription for Notch target genes (Hes-1 and Hey-1). The activation of the Notch signaling pathway facilitates tumor proliferation and metastasis; conversely, inhibition of this pathway eradicates CSCs and enhances drug sensitivity.^283^ The role of Notch as a regulator in CSCs and tumor microenvironment has been extensively studied, confirming both its positive and negative effects.^284^ Recent studies have confirmed through *in vivo* screening that at least four DUBs (BAP1, eIF3F, eIF3H, and USP10) exert regulatory control over Notch signaling in breast cells via the Ub proteasome pathway.^285^ The activation of the Notch developmental signaling pathway in triple-negative breast cancer is a characteristic feature, resulting in the secretion of pro-inflammatory cytokines and the recruitment of tumor-associated macrophages to the tumor microenvironment. The study revealed that USP9X, a deubiquitinase, forms a multiprotein complex with tribbles homolog 3, a pseudokinase, under cellular stress conditions commonly found in the tumor microenvironment and jointly activates the Notch pathway. The knockdown of USP9X abolishes Notch activation and reduces the production of pro-inflammatory cytokines, such as CCL2 and IL-1β. These molecular changes are accompanied by a reduction in tumor inflammation, enhancement of anti-tumor immune responses, and suppression of tumor growth.^286^ A separate study has demonstrated that both mammalian USP12 and its *Drosophila melanogaster* homologue serve as novel negative regulators of Notch signaling. Specifically silencing USP12 impedes the transport of Notch to lysosomes, resulting in an increase in cell surface receptors and heightened Notch activity. At the biochemical level, USP12 and its activator USP1-associated factor 1 catalyze the deubiquitination of the inactive form of Notch in cell culture and *in vitro*.^287^

### Ubiquitination in the Hh signaling pathway

The Hh signaling pathway is recognized for its pivotal role in orchestrating cellular growth, proliferation, and differentiation during embryonic development. Hh signaling pathway regulates cancer cell mesenchymal stem cell self-renewal and cancer tissue homeostasis.^288^ Hh signaling is transduced through a receptor complex consisting of Ptch and Ihog.^289^ When extracellular Hh ligands such as Sonic (SHh), Indian (IHh), or Desert (DHh) Hedgehog bind to PTCH, the inhibitory effect of PTCH on Smo is reduced, whereby Gli is translocated to the nucleus and induces the transcription of target genes.^290^ Aberrant activation of the Hh signaling pathway is an important driver of breast cancer, prostate cancer, non-small cell lung cancer, gastric cancer, and hematopoietic malignancies.^291^ The disruption of Smo, a key component in the Hh signaling pathway, resulted in the suppression of leukemic stem cells expressing BCR-ABL and led to an extended survival period in a mouse model of chronic myeloid leukemia research.^292^ An active Hh signaling pathway has also been identified in glioblastoma CSCs, and inhibition of pathway components through cyclobutamine or siRNA leads to a loss of tumorigenic potential.^293^ In breast cancer, the activation of the pathway in CSCs expressing GLI1 or GLI2 through Hh ligands or inhibition of GLI1 or GLI2 using cyclobutrazine or siRNA results in a change in BMI-1 expression. BMI-1 is a central regulator of self-renewal in normal stem cells and has tumorigenic potential both *in vitro* and *in vivo*.^294^ In addition, the Hh signaling pathway has been demonstrated to regulate the stemness of CSCs through the Ub proteasome. For instance, USP37 exhibits high expression levels in breast CSCs and is correlated with unfavorable prognosis among patients diagnosed with breast cancer. The USP37 protein is capable of modulating stemness, cellular invasion, and EMT via the Hh signaling pathway, while down-regulation of USP37 enhances the sensitivity of breast cancer cells to cisplatin 295. In particular, the genetic deletion of USP37 in these cells was found to result in a reduction of key components (SMO and GLI1) involved in the Hh signaling pathway, as well as stem cell markers (aldehyde dehydrogenase 1 and OCT4), at the protein level. Conversely, the activation of HH signaling induced by the agonist purmorphamine results in an up-regulation of USP37 gene expression, subsequently leading to the stabilization of GLI1 and influencing EMT in breast CSCs.^295^ Furthermore, Zhou et al discovered that USP48, a deubiquitinase enzyme, activates the Hh signaling pathway by enhancing the stability of the Gli1 protein in glioma cells.^296^ Through its C-terminal DUSP structural domain, USP48 interacts with Gli1 and removes Ub molecules from it. Knockdown of USP48 leads to the inhibition of Gli1 target gene expression, thereby suppressing cell proliferation and tumorigenesis. The intriguing finding is that Gli1 transcriptionally activates USP48 in glioma cells, thereby establishing a positive feedback loop that governs Hh signaling. In human glioblastoma, there exists a positive correlation between the expression levels of USP48 and Gli1. Moreover, high expression levels of USP48 are associated with a higher degree of malignancy in gliomas.^296^ These findings suggest that the regulatory axis involving USP48 and Gli1 plays a critical role in both glioma cell proliferation and glioblastoma tumorigenesis. The findings provide further evidence for the involvement of Hh signaling in the maintenance of stem cells and EMT,^295^ as well as the impact of DUB dysregulation on these oncogenic processes.^297^ In recent years, numerous studies have reported that dysregulation of the Hh signaling pathway is associated with a wide range of cancers. As a result, targeting this pathway has emerged as an attractive strategy for cancer therapy. The identification of diverse E3 ligases and deubiquitinases involved in the regulation of Hh signaling, along with the elucidation of their roles in cancer, has paved the way for the development of innovative therapeutic strategies to combat Hh-associated malignancies.^298^ In this context, certain E3 ligases and DUBs are emerging as promising therapeutic targets in various Hh-related tumors due to their active involvement in Hh signaling.^299^ Nevertheless, the development of more efficient and selective inhibitors of E3 ligases and DUBs for anti-cancer therapy while preserving normal physiology remains a formidable challenge.

### Ubiquitination in the Hippo-YAP signaling pathway

Given that CSCs are a key driver of tumorigenesis, and the role of Hippo signaling in CSCs has been extensively studied, our focus is on understanding how the Hippo signaling pathway regulates CSCs through the UPS. The Hippo pathway is well-known for its involvement in controlling CSC self-renewal, EMT, and the development of drug resistance. Activation of the Hippo pathway leads to phosphorylation and activation of LATS1/2 by the mammalian sterile 20-like kinase 1/2, which subsequently inhibits the activity of YAP/TAZ and prompts their translocation to the cytoplasm. This process suppresses TEA domain transcription factor-mediated gene expression, ultimately impeding CSC progression.^300^ Conversely, when Hippo signaling is suppressed, YAP/TAZ activation occurs, conferring CSC-like characteristics to cells and fostering tumorigenesis.^301^

Emerging evidence suggests that post-translational modifications play a pivotal role in mediating the activation and subcellular localization of signaling components, thereby modulating the induction, augmentation, or suppression of the corresponding signaling pathways. In this context, the Hippo pathway stands out as an exemplar, subject to various post-translational modifications including phosphorylation, acetylation, methylation, and ubiquitination. Disturbances in these modifications have been associated with the initiation and progression of tumorigenesis.^302^ Consequently, a focused exploration of the interplay between the Hippo pathway and ubiquitination in tumorigenesis and its advancement is warranted. Our discussion delves into the regulatory dynamics of ubiquitination-deubiquitination within the Hippo signaling pathway, recent strides in identifying therapeutic targets and strategies, and the prospective avenues that could contribute to more effective tumor diagnosis and treatment. Notably, key components of the Hippo pathway, such as LATS1/2 YAP/TAZ, angiomotin, and vestigial-like, have garnered considerable attention due to their pivotal roles. Both LATS1 and LATS2 proteins feature distinctive domains rich in proline, with the latter also exhibiting a PPxY motif. Phosphorylation, autophosphorylation, and ubiquitination sites are dispersed across both proteins.^303^ These proteins are central in governing cell fate, their influence spanning cell cycle, apoptosis, migration, and EMT processes, with implications for both oncogenesis and tumor suppression.^304^ A central tenet is the tumor suppressor role of LATS1/2 in curbing YAP/TAZ activity via the canonical Hippo pathway.^305^

In recent years, ubiquitination has emerged as a post-translational modification mechanism that is shared by both LATS1 and LATS2 proteins. Specifically, members of the NEDD4-like Ub ligase family interact with LATS1/2, thereby modulating Hippo pathway activity and tumorigenesis. Furthermore, the E3 Ub ligase ITCH governs the stability of LATS1, a serine/threonine kinase in the Hippo pathway, through a protein–protein interaction involving the PPxY motif of LATSI and the WW domain of ITCH. The ubiquitination of LATS1 by ITCH promotes its proteasomal degradation, leading to increased cell growth, epithelial–mesenchymal transition induction, and tumorigenicity. Conversely, depletion of ITCH results in elevated levels of LATSI, enhanced FAS-induced apoptosis, and reduced proliferation, survival, and migration.^306^ Likewise, neurofibromatosis type 2 hinders nuclear E3 Ub ligases CRL4^DCAFI^ activities of the RING finger subfamily, thereby maintaining LATS1 stability and preventing LATS2 ubiquitination-induced conformational change. These ligases involve key constituents like CUL4A, Rocl/Rbx1, DDB1, and DCAF1.^307^ Similarly, the E3 ligase SIAH2 governs LATS2 stability, impacting both Hippo and HIF signaling pathways, further underscoring hypoxia's role in modulating Hippo pathway ubiquitination.^308^ This exploration underscores the centrality of ubiquitination in shaping the Hippo signaling pathway's regulatory mechanisms, with implications spanning hypoxic conditions, tumor development, and potential therapeutic interventions.

Furthermore, the Hippo pathway's pivotal functional output is mediated through YAP and TAZ, acting as indispensable connectors and integrators of diverse signaling pathways, encompassing Wnt, G protein-coupled receptors, epidermal growth factor, and the Notch pathway.^309^ TAZ, sharing approximately 50% sequence identity with YAP,^310^ exhibits significant structural similarities. The WW domain, which contains two conserved tryptophan residues in identical positions, stands out as a hallmark feature in both YAP and TAZ, instrumental in bestowing signaling specificity by orchestrating the localization and activity of YAP/TAZ.^311^ Accumulating evidence strongly supports the concept that YAP and TAZ function as oncogenes in mammalian cells. Overexpression of YAP/TAZ in normal epithelial cells not only promotes cellular transformation but also confers a CSC phenotype.^312^ In murine models, up-regulation of YAP levels significantly enhances tissue hyperproliferation and precipitates carcinogenesis in various epithelial tissues.^313^ Moreover, it is noteworthy that aberrant activity of other signaling pathways, such as the GTPase KRAS pathway,^170^ can potentially impact the functionality of YAP and TAZ, thereby playing a critical role in tumor development. Much like LATS, YAP, and TAZ are under the sway of direct ubiquitination and deubiquitination. The FBXW7 gene is recognized as a tumor suppressor in human cancers, with decreased expression of FBXW7 being associated with unfavorable clinicopathological characteristics.^314^ It exerts its effects by inducing apoptosis and growth arrest, partly through targeting YAP ubiquitination and degradation in liver cancer.^315^ Another study unveils SOCS6's role as a recognition factor for YAP, orchestrating its degradation through the Elongin B/C Cullin-5 Ub ligase complex. Under Ras-induced down-regulation of SOCS5/6, YAP stability increases, highlighting the interplay between Ras and Hippo pathways. Ras-mediated cellular transformation hinges on the suppression of SOCS6-mediated YAP turnover, emphasizing YAP's integration point for Hippo and Ras pathways opposing activities.^316^ The RAS/mitogen-activated protein kinase pathway additionally functions by phosphorylating Ajuba to deactivate LATS kinase, resulting in reduced YAP phosphorylation, SCF-β-Trcp-dependent degradation of YAP protein, and enhanced YAP activity.^317^ RAS exerts its oncogenic role at least partially through these mechanisms. In the context of deubiquitination studies, a recent investigation has revealed that USP9X specifically targets YAP1 for deubiquitination and stabilization, thereby facilitating breast cancer cell survival and progression. The up-regulation of YAP1 degradation upon elimination of USP9X enhances cellular sensitivity to chemotherapy, indicating the oncogenic role of USP9X and identifying it as a potential therapeutic target for breast cancer treatment.^318^

**Figure 7:**
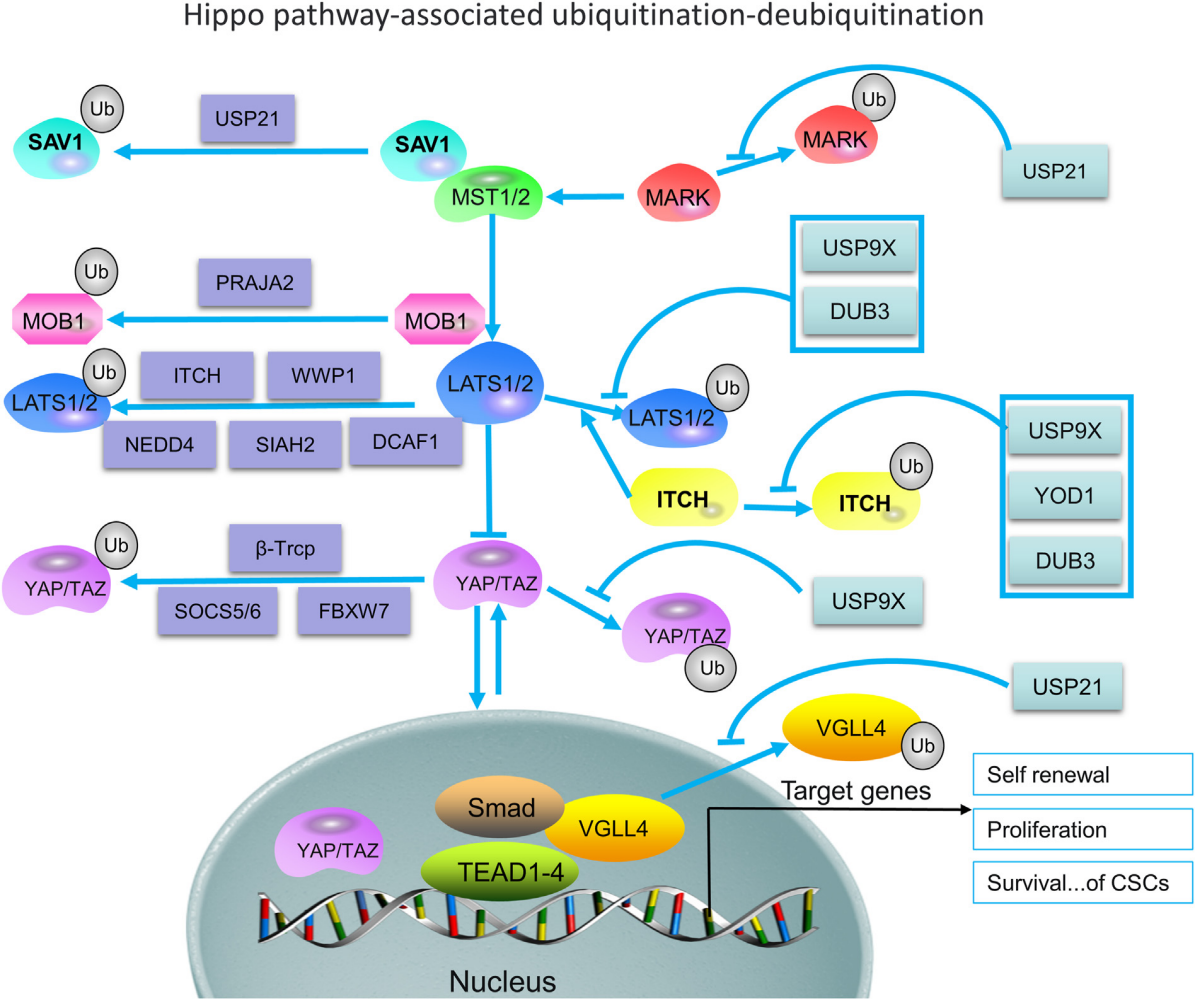
Hippo pathway-associated ubiquitination and deubiquitination.

The ubiquitination and deubiquitination processes of the Hippo signaling pathway are widely observed and represent important post-translational modifications, with numerous E3 ubiquitinases and deubiquitinases playing critical roles. Numerous components within the Hippo pathway are subject to regulation by both these post-translational modifications, and any dysregulation can contribute to the initiation and progression of tumorigenesis as well as metastasis. The efficacy of YAP and TAZ inhibition in the treatment of various cancer types susceptible to YAP/TAZ activation has been demonstrated by multiple studies.^319^ The Hippo pathway exhibits a complex network of crosstalk with other signaling pathways, which may also be regulated by ubiquitination-deubiquitination processes, thereby potentially influencing the Hippo pathway indirectly (Fig. 7). In addition, the development and progression of cancer involve numerous intricate biological processes, some of which remain elusive. All these facts contribute to the intricacy of tumorigenesis. Given that the study of Hippo pathway-associated ubiquitination-deubiquitination is still in its nascent stage, further research in this domain will undoubtedly contribute significantly to advancements in cancer diagnosis and treatment.

### Emerging drugs targeting CSCs through the regulation of ubiquitination

CSCs represent a minor subset of cells within tumors, possessing stem cell properties and exhibiting the ability to self-renew and differentiate, thereby sustaining tumor growth and metastasis. The existence of CSCs poses a significant challenge in cancer therapy due to their robust resistance to conventional chemotherapy and radiotherapy. The regulation of ubiquitination is a crucial cellular process that plays a role in various biological processes, including the cell cycle, DNA damage repair, and inflammatory response. Additionally, the growth and proliferation of CSCs are closely linked to ubiquitination. In recent years, numerous studies have demonstrated that small molecule inhibitors can effectively impede the growth and proliferation of CSCs by modulating the crucial ubiquitination process, thus providing valuable insights for preclinical investigations. This section will specifically address three aspects of ubiquitination in the regulation of CSCs, including investigational drugs targeting ubiquitinating enzymes, signaling pathways of CSCs, and epigenetic regulation. For example, MDM2 proteins can bind to p53 proteins and promote ubiquitination and degradation of p53, thereby reducing p53 stability and activity.^320^ The growth and proliferation of CSCs can be modulated by MDM2 inhibitors through the regulation of ubiquitination. The MDM2 inhibitors specifically hinder the interaction between MDM2 and p53, thereby enhancing the stability and functionality of p53 and facilitating p53-mediated ubiquitination. This ubiquitination promotes apoptosis and inhibits the proliferation of CSCs. In addition, MDM2 inhibitors can directly affect the ubiquitination process of CSCs, thereby regulating the growth and proliferation of CSCs. Therefore, MDM2 inhibitors could be potential drugs for the treatment of CSCs.^321^ However, the specificity of the MDM2-p53 protein interaction poses considerable difficulties in the development of small-molecule inhibitors, resulting in only a few candidates being in clinical trials (Table 2) and no marketed drugs targeting MDM2.

**Table 2:** Small molecule inhibitors of MDM2 in clinical trials.

Inhibitor	Structure	Phase	Combination of drugs	Tumor	Clinical trial No.
HDM201		I	Spartalizumab	Colorectal cancer, nonsmall cell lung carcinoma, triple-negative breast cancer, renal cell carcinoma, acute myeloid leukemia	NCT02890069
		Ib/II	Ribociclib	Liposarcoma	NCT02343172
		I	LXS196	Uveal melanoma	NCT02601378
MK-8242		I	Cytarabine	Acute myeloid leukemia	NCT01451437
		I		Solid tumors	NCT01463696
			
SAR405838		I	Pimasertib	Neoplasm malignan	NCT01985191
			
			
			
			
			
APG-115		Ib/II	5-Azacitidine	Acute myeloid leukemia	NCT04358393
		Ib	Azacitidine, Cytarabine	Acute lymphocytic leukemia, neuroblastoma, acute myeloid leukemia,	NCT04275518
		Ib/II	Pembrolizumab	Metastatic melanomas, advanced solid tumors	NCT03611868
CGM-097		I		Advanced solid tumors, TP53wt	NCT01760525
			
			
			
			
			
AMG232		I		Acute myeloid leukemia, advanced solid tumors, multiple myeloma	NCT01723020
		I	Radiation therapy	Brain cancer	NCT03107780
		Ib	Radiation therapy	Soft tissue sarcoma	NCT03217266
		Ib/IIa	Dabrafenib, Trametinib	Metastatic melanoma	
DS-3032b		I	5-Azacitidine	Acute myeloid leukemia, myelodysplastic syndromes	NCT023199369
				lymphomas	
			
			
		I/II	Cytarabine, Venetoclax	Refractory and relapsed acute myeloid leukemia	NCT03634228
			
RG7112		Ib	Doxorubicin	Sarcoma	NCT01605526
		Ib	Cytarabine	Acute myeloid leukemia	NCT01635296
		I		Solid tumors	NCT01164033
			
			
RG7388		III	Cytarabine	Refractory and relapsed acute myeloid leukemia	NCT02545283
		I/II	Atezolizumab	Breast cancer	NCT03566485
		I/II	Cyclophosphamide, Topotecan, Fludarabine, Cytarabine	Acute myeloid leukemia, acute lymphocytic leukemia, neuroblastoma, solid tumors	NCT04029688

In addition, DUB inhibitors represent a promising class of novel anti-tumor agents that have undergone extensive investigation for the treatment of various types of cancers. Recently conducted studies have demonstrated the significant potential of DUB inhibitors in specifically targeting CSCs. DUBs play a crucial role in maintaining the stemness of cancer cells by modulating various factors and signaling pathways associated with stem cell characteristics. The interventions aimed at DUBs consistently decrease the population of CSCs and alleviate the development of treatment resistance (Table 3). For instance, the deubiquitinating enzyme USP7 regulates the function of the MDM2-p53 complex and plays a crucial role in cancer diseases. The knockdown of USP7 suppresses the proliferation of colorectal cancer cells with varying p53 status, while inhibition of USP7 using its inhibitor P5091 attenuates Wnt signaling activity by promoting ubiquitination and subsequent degradation of β-catenin 322. The efficacy of DUB inhibitors surpasses that of proteasome inhibitors in the treatment of refractory tumors. Furthermore, b-AP15, a selective DUB inhibitor, demonstrates the ability to overcome resistance to bortezomib in patients with multiple myeloma.^323^ Eichhorn et al found that the high expression of USP15 in glioma tumors exerts oncogenic effects by activating the TGF-β signaling pathway. Inhibition of USP15 expression reduces the oncogenic capacity of patient-derived glioma-initiating cells.^324^ Additionally, a separate study has also established a correlation between USP1, USP7, and USP22 with glioma stemness due to their ability to enhance the protein stability of inhibitor of DNA binding 1 and Lysine (K)-specific demethylase 1A respectively.^194^^,^^325^^,^^326^

**Table 3:** Preclinical application of deubiquitinating enzyme inhibitors in targeted therapy for cancer stem cells.

Deubiquitinating enzyme	Inhibitor	Structure	Mechanism	Cancer stem cell type	References
USP14	VLX1570		Regulates the progression of epithelial–mesenchymal transition and induces cell apoptosis	Gastric cancer, multiple myeloma	327,328
USP1	Pimozide		USP1 promotes the stability and stem-like characteristics of ID proteins in osteosarcoma	Glioma, osteosarcoma	210,325
USP7	P5091		USP7 inhibits LSD1 ubiquitination, stabilizes LSD1, causes G0/G1 blockade, and promotes tumorigenesis and invasion	Glioma	194
USP9X	WP1130		The degradation of p53 by USP9X can be inhibited by WP1130, thereby enhancing the stability of p53 and suppressing the development and progression of hepatocellular carcinoma	Hepatocellular carcinoma, glioblastoma	329,330

In summary, DUB inhibitors can affect the growth and proliferation of CSCs by regulating protein ubiquitination. Specifically, DUB inhibitors can inhibit the activity of DUBs, thereby increasing the level of protein ubiquitination and promoting protein degradation and apoptosis. In addition, DUB inhibitors can affect the signaling pathways of CSCs, such as Wnt and Notch, thereby regulating the proliferation and differentiation of CSCs. Therefore, the investigation of inhibitors targeting ubiquitination is crucial for effectively suppressing the growth and proliferation of CSCs. This not only provides new insights and directions for the treatment of CSCs but also provides innovative strategies and methods for tumor therapy.

## Conclusions and future perspectives

Ubiquitination has emerged as a well-established mechanism for the regulation of signal transduction, and the Ub system is intricate, multifaceted, and indispensable for governing numerous cellular processes. The process of ubiquitination is tightly regulated at multiple levels by a cascade of enzymes, including E1, E2, and E3 ligases, as well as by a series of DUBs. The UPS pathway governs protein degradation via the proteasome and exerts regulatory control over a diverse array of cellular processes, encompassing transcription factors, epigenetic regulators, pivotal oncoproteins, and proteins associated with stemness. The UPS has been demonstrated in numerous recent studies to exert control over a range of biogenesis processes, including tumor cell proliferation, differentiation, metastasis, and drug resistance through targeted degradation of proteins associated with tumor stem cells. Furthermore, ubiquitination is critical for the dynamic regulation of programmed cell death. The inherent drug resistance of CSCs, positioned as the “root” in tumors, presents a formidable challenge in conventional therapeutic strategies due to underlying signaling and epigenetic perturbations.

The dysregulated expression of numerous E3 Ub ligases and DUBs in CSCs can function as “negative seed cultivators” to maintain cancer stemness, thereby reinforcing their activity and establishing a vicious cycle. The latest research findings have revealed that a plethora of E3 Ub ligases and DUBs have been extensively characterized in various immune cell types, as well as cancer cells, serving as pivotal regulators of both anti-tumor immunity and tumor-induced immune suppression. The efficacy of cancer immunotherapy can be enhanced by targeting Ub-related factors in CSCs, as evidenced by an increasing number of preclinical studies. Considering the existence of over 600 E3s and approximately 100 DUBs in mammalian cells, Ub-dependent therapeutic approaches present significant prospects for drug development targeting the elimination of CSCs and cancer treatment. This article serves as a comprehensive guide to the regulatory intricacies surrounding Ub-associated receptors and ligand proteins within CSCs. The discussion encapsulates various entities such as Ub-related E3 ligases, deubiquitinases, stemness-associated transcription factors, and the regulatory interplay within Notch, Hh, Wnt/β-catenin, and Hippo-YAP signaling pathways in CSCs. Additionally, transcription factors play a pivotal role in the regulation of diverse cellular processes, including but not limited to cell proliferation, metastasis, EMT, CSCs, and resistance to chemotherapy. Dysregulated expression levels of transcription factors in CSCs contribute to tumorigenesis and malignant progression. Moreover, the expression of transcription factors is tightly regulated by multiple signaling pathways, noncoding RNAs, and E3 Ub ligases. Recently, studies have demonstrated that transcription factors involved in the regulation of CSC formation encompass Oct4, SOX2, KLF4, c-Myc, Nanog, GATA, split-like protein 4, Bmi-1, oligodendrocyte transcription factor 2, POU class 3 homeobox 2, and FOX proteins. The present review article delineates the pivotal roles of transcription factors associated with CSCs and elucidates the regulatory mechanisms underlying the degradation of these CSC-associated transcription factors by specific E3 ligases. Additionally, we provide a comprehensive overview of the Notch, Hh, Wnt/β-catenin, and Hippo-YAP signaling pathways implicated in the regulation of Ub proteases in CSCs. The involvement of Notch, Hh, Wnt/β-catenin, and Hippo-YAP signaling pathways has been extensively documented in various CSCs. The up- or down-regulation of Notch, Hh, Wnt/β-catenin, and Hippo-YAP signaling pathways is mediated by multiple signaling factors through post-translational modifications, and dysregulation of these pathways is associated with various human malignancies. Among the numerous post-translational modifications involved, ubiquitination and deubiquitination play crucial roles in regulating most signaling pathways such as Notch, Hh, Wnt/β-catenin, and Hippo-YAP. Therefore, a comprehensive investigation into the regulatory role of the UPS in CSC signaling will pave the way for novel clinical therapeutic applications and drug discovery against human diseases.

## Ethics declaration

The study protocol was approved by the Ethics Committee for Animal Experimentation of China Pharmaceutical University. Written informed consent was obtained from all participants prior to their inclusion in the study. The purpose, procedures, potential risks, and benefits of the study were explained to each participant, and they were allowed to ask questions before providing their consent. The authors confirm that they have obtained the necessary consent for publication from all individuals whose personal information, images, case details, or other identifiable data are included in the manuscript.

## Funding

This work was supported by grants from The 10.13039/501100001809National Nature Science Foundation of China (No. 82173842), WU JIEPING Medical Foundation (No. 320.6750.2023-05-7), and The Medical Science and Technology Research Project of Henan Province, China (No. SBGJ202003010).

## Author contributions

**Q.G.**, **H.Q.**, **Z.C.**, and **W.Z.** reviewed the literature and drafted the article. **L.Z.** and **T.Q.** finalized the manuscript and provided suggestions to improve it. All authors participated in designing the concept of this manuscript. All authors contributed to the article and approved the submitted version.

## Data availability

The data will be made available on reasonable request.

## Conflict of interests

The authors have no relevant financial or non-financial interests to disclose.
